# Artificial plateau neurons with in-situ spike-malleability for rhythmic quadrupedal locomotion

**DOI:** 10.1038/s41467-026-72428-2

**Published:** 2026-04-28

**Authors:** Hailiang Wang, Yishu Zhang, Qingao Chai, Qian He, Jiayang Hu, Yongqing Bai, Guanyu Liu, Zongwen Li, Jian Chai, Xin He, Mengze Zhao, Guodong Xue, Kaihui Liu, Yu Fu, Huajin Tang, Yang Xu, Bin Yu

**Affiliations:** 1 College of Integrated Circuits, Zhejiang University, Hangzhou, Zhejiang China; 2 ZJU-Hangzhou Global Scientific and Technological Innovation Center, Hangzhou, Zhejiang China; 3 College of Computer Science and Technology, Zhejiang University, Hangzhou, China; 4 State Key Lab of Brain-Machine Intelligence, Zhejiang University, Hangzhou, China; 5 State Key Laboratory for Mesoscopic Physics, Frontiers Science Center for Nano-Optoelectronics, School of Physics, Peking University, Beijing, China; 6 Key Laboratory of Quantum State Construction and Manipulation, Department of Physics, Renmin University of China, Beijing, China

**Keywords:** Electronic devices, Electronic devices, Motor control

## Abstract

Whole-body intelligent locomotion systems face persistent challenges of redundant actuation and poor energy efficiency, limiting real-world deployment. Bio-inspired central pattern generators offer a promising framework for rhythmic control, yet hardware implementations struggle to match the efficiency and adaptability of biological systems. Here, we introduce an in-situ spike-malleable artificial plateau neuron integrating a bistable plateau gate with a transient threshold-switch. The neuron generates amplitude-programmable rhythmic spike bursts, achieving energy-efficient, antagonistic activation of extensors and flexors via a scalable circuit comprising two paired units (plateau gate and threshold-switch). The design leverages distributed encoding for coordinated muscle control, operating at ultra-low energy dissipation (141.37 pJ/spike). An expanded four-unit circuit enhances dynamic spike malleability, enabling parallel processing for multi-joint coordination. On a quadruped robot (Unitree Go2), these distributed circuits directly drive joint-level proportional derivative controllers using the Gaussian-filtered rhythmic spikes, enabling energy-efficient trotting without centralized computation. Critically, the system achieves stable on-ground locomotion and demonstrates adaptive gait transitions in real-world environments. Our approach merges ultra-compact hardware with bio-inspired architecture, advancing neuromorphic systems for energy-efficient autonomous robotics.

## Abstract (web summary)

The authors report an artificial plateau neuron with bistable, spike-malleable dynamics that generates programmable rhythmic bursts, enabling a quadruped robot to walk adaptively and energy-efficiently via distributed hardware control without centralized computation.

## Introduction

Traditional robotic locomotion systems rely on centralized computational control to coordinate rhythmic joint movements, often resulting in motion redundancy and high energy consumption due to complex architectures and power-intensive actuators^1–3^. Biological central pattern generators (CPGs), in contrast, employ distributed neural oscillators to dynamically regulate rhythmic patterns for locomotion, respiration and mastication with remarkable efficiency^4–7^. These networks leverage rotational dynamics to minimize redundancy while enabling multimodal motion, offering a blueprint for energy-efficient robotic control^8,9^. Recent advances in bioinspired coupled-oscillator systems have sought to replicate CPGs' functionality by coordinating peripheral neuromorphic circuits^10–13^. However, such approaches suffer from temporal latency and energy overheads due to the decoupling of signal generation and processing, as well as an inability to maintain in-situ memory states—critical to adaptive rhythmic control^9,14–17^. Realizing CPG-like efficiency in hardware, therefore, remains a key challenge.

**Fig. 1:**
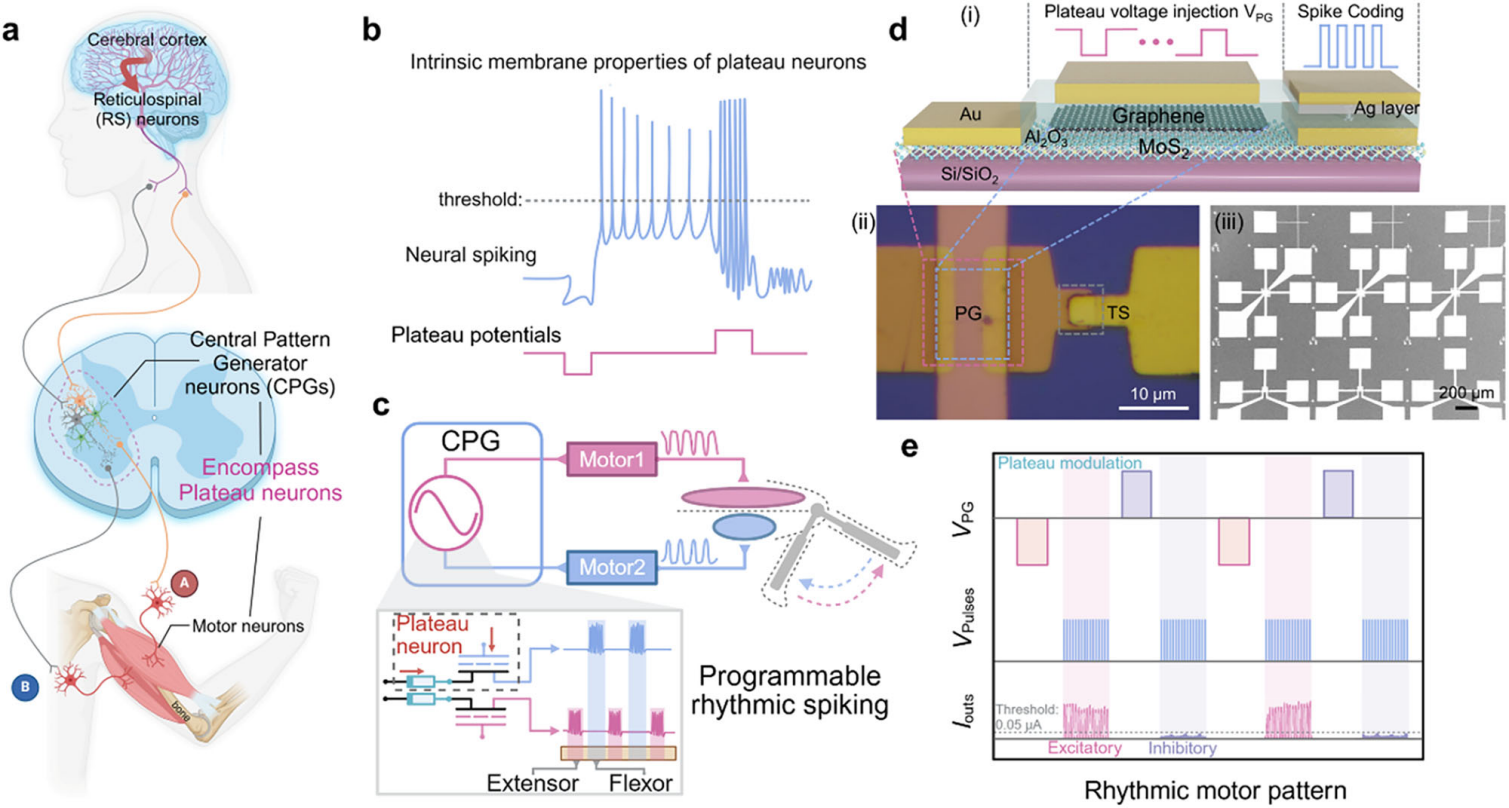
Artificial plateau neurons. **a** Illustration of neural signaling pathways underlying human locomotion. **b** Membrane potential firing characteristics of plateau neurons. A depolarizing pulse evokes sustained potentials in plateau neurons that persist after depolarization offset. These potentials can be terminated by a hyperpolarizing pulse. **c** Biomimetic CPGs antagonistic rhythms in motor control via a plateau neuron-based dual-branch circuit. **d** Schematic of the artificial plateau neuron device with PG-TS structure (i). Optical microscope image (ii) showing local *V*_PG_-modulated, TS stimulus applied, and 10 μm channel length. Scanning electron microscopy (SEM) image of 2(PG-TS) and 4PG-TS circuit structures (iii) on the 2-inch wafer as presented in Fig. 3a. **e** Plateau potentials in PG-TS enable in-situ plasticity that, in conjunction with upstream inputs, facilitates the encoding of rhythmic motor patterns. Negative *V*_PG_ maintains PG-TS in persistent excitation, generating sustained spike outputs in response to *V*_pulses_ inputs. Conversely, positive *V*_PG_ induces persistent inhibition. Rhythmic encoding of these *V*_PG_ enables the neurons to produce corresponding rhythmic spike train outputs (spike threshold: 0.05 µA). **a**, **b** created in BioRender. Hailiang Wang. (2026) https://BioRender.com/y56j682.

The human musculoskeletal system provides a compelling model for addressing these limitations (Fig. 1a). Here, the cerebral cortex coordinates CPGs, which act as rhythm generators for muscle activity through specialized plateau neurons (see Grillner^18^ and K. Eckstein^19^ for other plausible architectures). These neurons exhibit bistable behavior (resting hyperpolarized and active depolarized states), sustained depolarization, and intrinsic rhythmicity—enabling energy-efficient, self-sustaining oscillations without continuous external input (Fig. 1b)^20–23^. While various artificial neurons, such as spike-driven visual neurons^24,25^, organic electrochemical neurons^26–28^, and thermally Mott-neurons^29^ have advanced neuromorphic computing, they lack the in-situ spike-malleability required for dynamic CPG-like rhythmic encoding. For instance, neurosynaptic devices relying on shared input terminals for stochastic spiking and synaptic feedback^30,31^ fail to achieve the sustained depolarization of bistability essential for locomotion control.

In this work, we introduce an in-situ spike-malleable plateau neuron (PG-TS) that integrates a bistable plateau gate (PG) and a volatile threshold switching (TS) to emulate biological CPGs’ rhythmic dynamics. Fabricated using a single-layer chemical vapor deposition (CVD) molybdenum disulfide (MoS_2_) with a single-layer CVD graphene floating gate, the PG-TS neuron generates amplitude and phase programmable rhythmic spike bursts under plateau voltage modulation. This design eliminates the need for continuous biasing, significantly reducing power consumption. To demonstrate scalability, we design a bibranched circuit topology termed the 2(PG-TS) neuron circuit in which dual PG-TS units synergistically regulate antagonistic spike dynamics (Fig. 1c). This circuit leverages complementary modulation of *V*_PG1_ and *V*_PG2_ to generate the CPG-like, plausible, rhythmic antagonistic activations of extensor and flexor muscles, achieving an energy consumption of only 141.37 pJ/spike at 500 Hz, comparable to the energy dissipation levels of biological neural activity. Further, we design a four-channel PG parallel modulation circuit (4PG-TS) that expands the dynamic plasticity range of the plateau neuron output spikes by computational demonstrations of multiply-accumulate (MAC) operations. The 2(PG-TS) and 4PG-TS circuits, fabricated on a 2-inch wafer, showcase the potential of bioelectronic systems to achieve sophisticated, bioinspired rhythmic neural encoding and signal processing capabilities.

We demonstrate a neuromorphic hardware approach to autonomous quadrupedal locomotion, implementing distributed 2(PG-TS) rhythmic oscillators that directly synthesize amplitude- and phase-programmable rhythmic spiking bursts for robot motor control. Unlike those common position-based methods, our rhythmic oscillators modulated by plateau voltage (*V*_PG_) generate antagonistic flexor/extensor spikes with 180° anti-phase synchronization, enabling energy-efficient diagonal-pair gait coordination. The Gaussian-filtered spike-to-angle conversion drives proportional-derivative (PD) controllers in the Unitree Go2 robot, achieving closed-loop, end-to-end motor control without centralized computation. We validate stable trotting through simulations (Isaac Gym) and physical experiments, including on-ground walking and adaptive gait modulation via external inputs, with programmable spiking dynamics enabling rhythmic locomotion. This hardware-centric paradigm establishes a scalable framework for deployable neuromorphic robotics. This research work bridges the biological principles of rhythmic motion with neuromorphic hardware implementation, offering a viable pathway toward adaptive, energy-autonomous robotic locomotion.

## Bio-inspired artificial plateau neuron

Biological plateau neurons, or pacemaker neurons, are distinguished by their ability to sustain plateau potentials, exhibiting adaptive spiking patterns and displaying bistable, hysteretic membrane potential dynamics^20,23,32^. These features make them ideal for various rhythmic control and pattern generation functions in neural circuits. Inspired by these capabilities, we design a unique structure that leverages plateau potential control, comprising a PG-controlled MoS_2_/Al_2_O_3_/Graphene/Al_2_O_3_ stack to inject the plateau voltage. The drain integrates a volatile integrate-and-fire TS (Ag/Al_2_O_3_/Au) component that encodes spikes via continuous voltage pulse input (Fig. 1d (i)). This architecture utilizes non-volatile hyperpolarization and depolarization to modulate the synaptic weights of the PG-TS, enabling spatiotemporal encoding in plateau neurons. The classical two-dimensional (2D) floating gate non-volatile weight adaptive modulation architecture facilitates sustained depolarization and spike-adaptive capabilities. The PG erase and program operations achieve bistable modulation of neuronal depolarization and hyperpolarization. Figure 1d (ii) and (iii) show an optical microscope image of the top-view of the artificial plateau neuron PG-TS device and a scanning electron microscope (SEM) image of the extended plateau neuron circuits on a 2-inch wafer, including the 2(PG-TS) and 4PG-TS configurations. The detailed fabrication process for the PG-TS, 2(PG-TS), and 4PG-TS structures is illustrated in Supplementary Figs. 1. Notably, all devices are fabricated using the same flow, demonstrating the feasibility of large-scale production and circuit-level integration of plateau neurons. The structural and morphological properties of single-layer CVD MoS_2_ and single-layer CVD graphene are thoroughly characterized using Raman spectroscopy and atomic force microscopy (AFM) analysis (Supplementary Figs. 2, 3, and 4). As shown in the schematic (Fig. 1e), the PG-TS neuron maintains an active state after a negative *V*_PG_ pulse (depolarization). Under continuous upstream stimulation, this active state results in persistent spike bursts. Conversely, a positive *V*_PG_ pulse (hyperpolarization) holds the neuron in a quiescent state, effectively shutting off spiking even during ongoing upstream excitation. The bistable control and persistent depolarization of PG-TS enable the generation of adaptive, rhythmic locomotion control signals directly at the device level, highlighting its potential for highly integrated, low-power intelligent motion control systems.

## Spatiotemporal dynamics of the plateau neuron

**Fig. 2:**
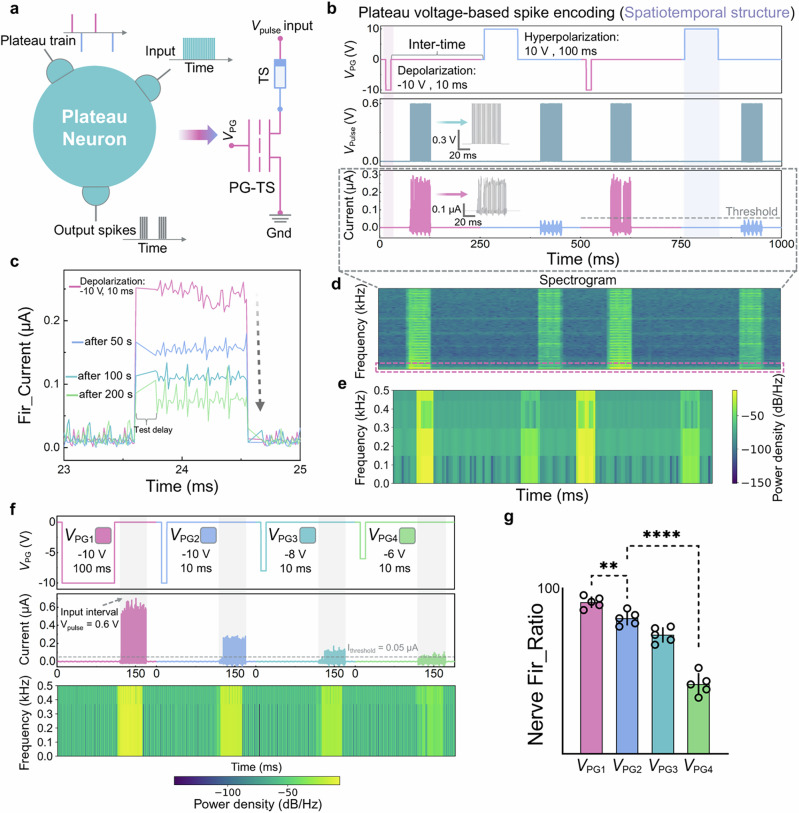
Demonstration of spike-malleable dynamics in PG-TS. **a** Schematic of plateau neuron with an in situ spike-malleable and PG-TS unit. The plateau-train corresponds to the *V*_PG_ gate. **b** Spatiotemporal encoding of plateau voltage-based spikes in PG-TS. With *V*_PG_ (10 V, 10 ms) programming, the PG-TS neuron generates a persistent spiking response by integrating incoming pulses (*V*_Pulse_ = 0.6 V, *T*_interval_ = 1 ms, duty-cycle = 50%). Conversely, *V*_PG_ (−10 V, 100 ms) erase deactivates the PG-TS neuron completely. The inset gray trace provides zoomed views of the detailed spike waveforms. **c** Temporal dynamics of persistent spikes extracted from activated PG-TS. **d**, **e** Frequency domain spectrogram (d) corresponds to the PG-TS spike waveform in (**b**), generated via a (plt.specgram()) function in Python. The PSD heatmap of the frequency domain signal following low-pass filtering (**e**). The filtering process was employed to remove high-frequency noise components while retaining the frequency band corresponding to the rhythmic spiking of PG-TS neurons, thereby providing a clear visualization of the rhythmic in PG-TS event-driven operation. **f**, **g** Persistent plasticity of PG-TS neuron spike output PSD (f) and nerve fir_ratio (**g**) under varying *V*_PGi_ stimulation (*i *= 1, 2, 3, 4).

The spatiotemporal dynamics of the PG-TS device are illustrated in Fig. 2a, which depicts the signal flow of the plateau neuron and the circuit topology of the PG-TS device. Excitatory pre-inputs to the plateau neuron undergo rhythmic plateau-train modulation, resulting in a rhythmic spike signal. The PG-TS device closely matches the biological signal flow structure of plateau neurons^4,20^. Specifically, volatile TS receives upstream excitation (voltage pulse input) to generate spiking output (Supplementary Fig. 5)^33–35^. The spiking fire rate of the integrate-and-fire TS component increases significantly with enhanced input pulse amplitude (Supplementary Fig. 6). The static energy band distribution within the PG structure and the dynamic evolution of the energy band during the non-volatile configuration switching process are illustrated in Supplementary Fig. 7^36^. The non-volatile PG is critical to enabling artificial plateau neurons to exhibit bistability and in-situ spike-malleability (Supplementary Fig. 8). Under rhythmic modulation of *V*_PG_, the PG-TS achieves in-situ spike-malleability, generating a serial rhythmic control signal. Supplementary Fig. 9 presents SEM images of the PG-TS device on a 2-inch wafer. The spike encoding under plateau voltage modulation in the PG-TS reflects the spatiotemporal structure of biological plateau neurons (Fig. 2b). Supplementary Fig. 10 details the continuous voltage pulse applied to the TS terminal. After depolarization (with *V*_PG_ = −10 V for 10 ms), the PG-TS enters an active state capable of integrating input pulses to produce sustained spikes. Conversely, hyperpolarization (with *V*_PG_ = 10 V for 100 ms) leads to complete deactivation of spikes. Further characterization of the depolarization and hyperpolarization dynamics in the PG-TS device is provided in Supplementary Fig. 11. The ability to reversibly toggle between active and quiescent states under *V*_PG_ modulation aligns with the behavior of biological plateau neurons. This mechanism allows rhythmic information associated with plateau voltage to be encoded across a temporal axis of spike sequences, resulting in rhythmic bursts that mimic biological locomotion signals^23,37^.

The nonlinear temporal dynamics of sustained depolarization-triggered spikes in the PG-TS device are depicted in Fig. 2c. With the upstream input pulse intensity held constant, the applied depolarization voltage triggers high-amplitude, persistent spikes that exhibit a nonlinear self-decay characteristic over time. This sustained depolarization is a core feature enabling biological plateau neurons to achieve low-power, adaptive rhythmic motor control^2,38^. The nonlinear relationship between sustained depolarization-triggered spikes in plateau neurons may also have potential applications in time-series training tasks or temporally correlated dynamic recognition, though this work primarily focuses on rhythmic locomotion control. Dynamic spike switching processes in the depolarized PG-TS device, along with mixed-mode spiking output, are illustrated in Supplementary Fig 12. Among hardware implementations for rhythmic locomotion, our latency of 5.62 µs is notably lower than other oscillator-based CPGs (e.g., VO₂-based systems operating at ms-scale) and software-based spiking network controllers (~10 ms) (Supplementary Table 1). This microsecond-level response provides a substantial performance margin well within the 10−100 ms control latency requirement for stable dynamic gait locomotion, enabling future exploration of high-bandwidth adaptive control^39,40^. Further characterization of the rhythmic signal frequency spectrum is shown in Fig. 2d. The power spectral density (PSD) heatmap in Fig. 2e, obtained after low-pass filtering, aims to remove high-frequency noise while preserving the frequency range (*f*_fir_ ≈ 0–0.5 kHz) associated with the rhythmic spike outputs. In the PSD visualization, the bright yellow regions indicate periods of neuronal spike bursts, while the greener areas represent quiescent states. The PSD is calculated using the formula:

1 $${{{{\rm{PSD}}}}}_{{{\rm{dB}}}/{{\rm{Hz}}}}=10{\log }_{10}\left({\left|X\left({f}_{{{\rm{signal}}}}\right)\right|}^{2}\right)/N\cdot \Delta f$$

where |X(*f*_signal_)|^2^ is the squared magnitude of the signal’s Fast Fourier Transform (FFT) at frequency *f*_signal_, *N* is the number of samples, and Δ*f* is the frequency resolution. This visualization characterizes the rhythmic bursting behavior in PG-TS’s spatiotemporal spike event outputs, consistent with the intermittent, rhythmic nature of biological locomotion control^41–43^. To evaluate the potential of the PG-TS with in-situ spike-malleable in modulating rhythmic motor control signals, we varied the stimulation strength of *V*_PGi_ (where *i* = 1, 2, 3, 4). This modulation revealed the capability for nonlinear adjustments of spike burst amplitude and PSD, corresponding to movement amplitude and velocity regulation, respectively (Fig. 2f). The modulation of spiking neuronal firing rates, which emulates dynamic probabilistic modulation capability, is presented in Fig. 2g. Each bar represents data from five PG-TS devices. The PG-TS device demonstrates the ability to modulate rhythmic motor control signals within a single neural pathway. However, to realize more diverse and complex locomotion patterns, further scalable plateau neuron circuit designs are necessary.

## Dual-channel rhythmic control with a bibranched 2(PG-TS) circuit

**Fig. 3:**
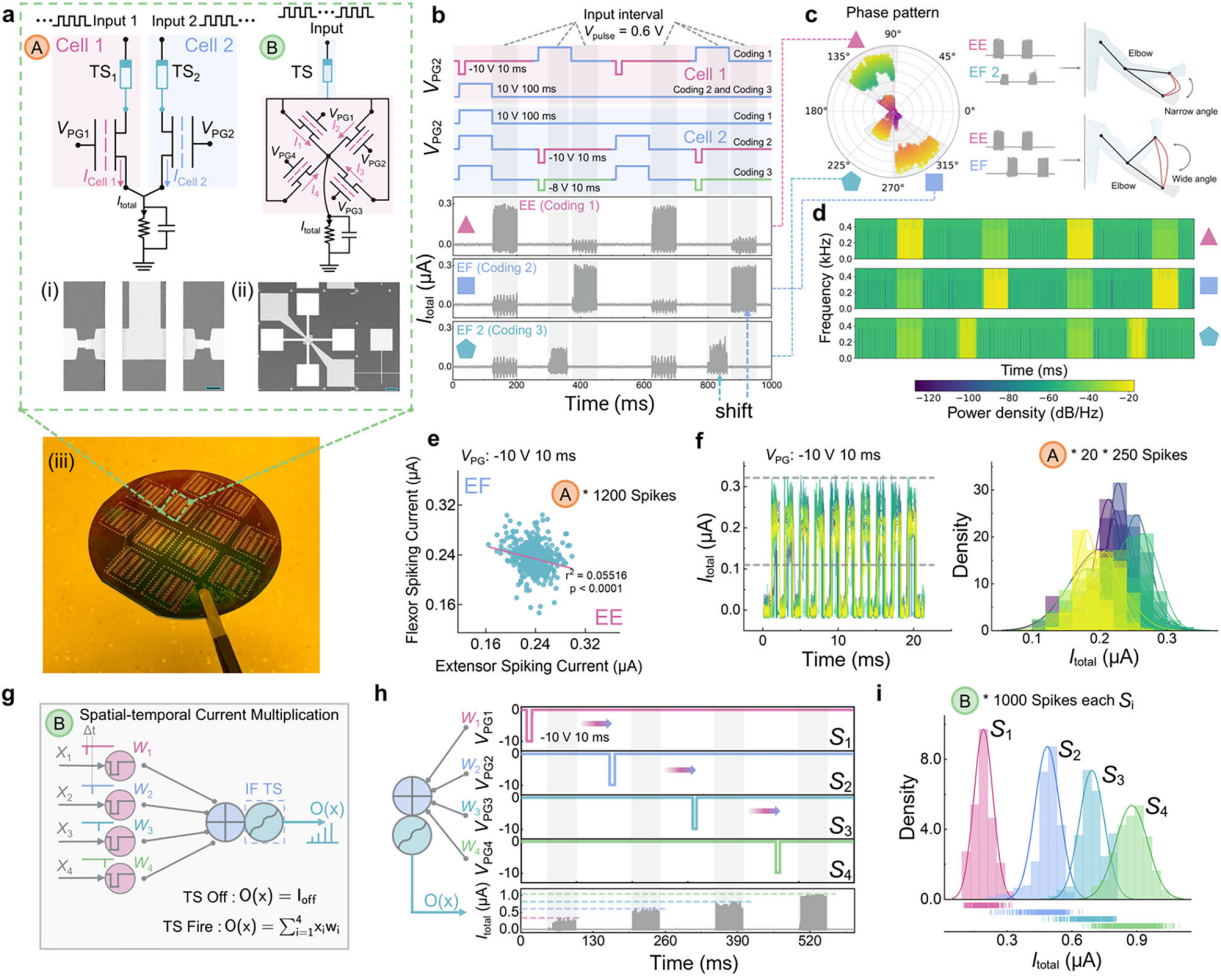
Demonstration of scalable plateau neuron circuit: 2(PG-TS), and 4PG-TS. **a** Schematic illustration of 2(PG-TS) with concurrent branches (A) and 4PG-TS with 4 parallel *V*_PGi_ control terminals (B). SEM images of 2(PG-TS) (i) and 4PG-TS (ii) circuit structures. A 2-inch semiconductor wafer showcasing the integrated fabrication of PG-TS, 2(PG-TS), and 4PG-TS constructs (iii). *I*_total_, total current of output. **b** Temporal output spike patterns of 2(PG-TS) neuromorphic circuit structure. The independent control of *V*_PG1_, and *V*_PG2_ enables 3 modulation modes (Coding 1, Coding 2, and Coding 3). Continuous input pulse train, shown by the shaded gray region (*V*_Pulse_ = 0.6 V, *T*_interval_ = 1 ms, duty-cycle = 50%). Pink triangle represents EE (Coding 1), blue square represents EF (Coding 2), and cyan pentagon represents EF 2 (Coding 3). EE elbow-extensor, EF elbow-flexor. **c** Phase pattern obtained from output spike patterns (EE, EF, and EF 2). **d** The frequency domain characteristics of neural spike events are further analyzed through PSD heatmap visualizations of the EE, EF, and EF 2 modulation modes after low-pass filtering. **e** Flexor spiking current (µA of FE) versus extensor spiking current (µA of EE) across 1200 spikes: linear regression, *p* < 0.0001, *r*^2^ < 0.05516. **f** A 200-spike schematic is compiled by extracting 10 sequential spikes from each of 20 2(PG-TS) samples; 250 spike currents extracted from 20 samples exhibited normal distributions. **g** Schematic of the spatial-temporal current multiplication in an artificial plateau neuron with a 4PG-TS circuit can be divided into two parts: The first part involves MAC calculations, while the second part encompasses the nonlinear spike generation (IF-TS). Here, *X*_i_ represents the input vector, *W*_i_ denotes the stored ar_i_thmetic weight, and O(x) is the output spike sequence (*i* = 1, 2, 3, 4). **h** Diagram of 4PG-TS circuit output characteristics. The 4PG-TS circuit’s plateau voltage control of *V*_PG1_ (*S*_1_), *V*_PG2_ (*S*_2_), *V*_PG3_ (*S*_3_), and *V*_PG4_ (*S*_4_) generates corresponding weights *W*_1_, *W*_2_, *W*_3_, and *W*_4_. The shaded gray region denotes the continuous input pulse domain (*V*_Pulse_ = 0.6 V, *T*_interval_ = 1 ms, duty-cycle = 50%). **i** Schematic of 4PG-TS circuit output spikes normal distribution analysis.

Biological limb locomotion relies on dual-channel rhythmic control of extensor and flexor, governed by phase-complementary rhythmic spikes from CPGs unit^18^. To replicate this mechanism, we design the bibranched 2(PG-TS) circuit in Supplementary Fig. 13. Figure 3a(A) depicts the detailed signal flow and circuit structure of the bibranched 2(PG-TS) circuit. The SEM image in panel (i) shows the physical structure of the 2(PG-TS) circuit, while panel (iii) highlights its potential for large-scale manufacturing and circuit-level integration of artificial plateau neurons (PG-TS). To address two distinct rhythmic amplitude control scenarios for the elbow joint, we implement three encoding modes on the 2(PG-TS) circuit: Coding 1, Coding 2, and Coding 3. Coding 1 encodes rhythmic spike bursts to control the contraction of the elbow extensor (EE). Coding 2 generates rhythmic contraction control signals for the elbow-flexor (EF), where *V*_PG_ encoding involves hyperpolarizing Cell 1 while rhythmically depolarizing Cell 2. Coding 3 utilizes weaker depolarization intensity and varied temporal *V*_pulse_, resulting in shifts in the amplitude (burst amplitude and firing probability) and phase of the EF actuation (Fig. 3b). The phase distributions of three representative rhythmic control signals (EE, EF, and EF 2) demonstrate that desired phase patterns can be obtained from the 2(PG-TS) by modulating the PG input depolarization and *V*_pulse_ distribution, without altering the network topology (Fig. 3c). The 2(PG-TS) structure allows for programming of the relative phase between the EE and EF 2 outputs, generating spike patterns closely resembling small-amplitude elbow-joint extensor and flexor activation. In contrast, the phase relationship between the EE and EF outputs enables simulation of large-angle elbow-joint swing. This ability to modulate relative phase underscores the multifunctionality of 2(PG-TS) in reproducing complex neurodynamic biological locomotion control^44^. The PSD heatmap visualization depicts the distribution of spike bursts in EE, EF, and EF, with the locomotion control rhythm encoded spatiotemporally in spike intensity and temporal distribution (Fig. 3d).

The dynamic distribution of the EE and EF spikes exhibits a high degree of symmetry (Fig. 3e), consistent with the observed symmetry in biological locomotion muscle control^4,17^. From symmetric spikes of the 1200 motion potentials in EE and EF, the average active transient-power dissipation *P*_mean_ is 141.37 nW at a frequency of 500 Hz. Neglecting quiescent power dissipation, the infinitesimal energy consumption per spike *E*_Spike_ is calculated to be 141.37 pJ/spike (Supplementary Note 1), approaching the active energy dissipation of the human neuromuscular system. We extracted depolarization spikes from 20 samples of the 2(PG-TS) circuit to characterize its uniformity (Fig. 3f). While the normal distribution curves for each sample do not overlap, the combined 5,000 spikes from these samples follow a macroscopic normal distribution. The compact and bioinspired 2(PG-TS) achieves dual-channel rhythmic locomotion control signal modulation with minimal power output, injecting new momentum into the development of area-efficient, low-power, adaptive locomotion control circuits.

## Scalable plateau neuron circuit: 4PG-TS

The rhythmic dynamic spike range is limited by the modulation capability of a single PG depolarization in the PG-TS. To address this limitation, we introduce neuromorphic MAC operations. Specifically, we design the 4PG-TS artificial plateau neuron structure shown in Fig. 3a (B), which uses four parallel PGs to achieve a wide range of spike modulation through MAC operations^45^. Figure 3g details the spatial-temporal current multiplication of the 4PG-TS structure. The signal is multiplied by the depolarized weights, summed according to Kirchhoff’s law, and subjected to nonlinear integrate-and-fire TS integration to generate the output spikes. By successively depolarizing the parallel PGs, the output produces stepped spike currents, significantly expanding the spike modulation capability of the artificial plateau neuron (Fig. 3h), in line with our design expectations. The gradual enhancement of spike phase stability (spike jitter amplitude/spike amplitude) through incremental parallel PGs depolarization demonstrates a robust and reliable method for producing high-fidelity rhythmic control signals (Supplementary Fig. 14). Figure 3i shows the normal statistical distribution of 1,000 output spikes, following stepwise depolarization of the four parallel PGs, illustrating the adjustable dynamic spike threshold and expanded spike range. The 4PG-TS circuit represents a combinatorial expansion of the artificial plateau neuron architecture, enabling it to meet a diverse array of rhythmic locomotion requirements.

## Rhythmic gait control in quadruped robots

**Fig. 4:**
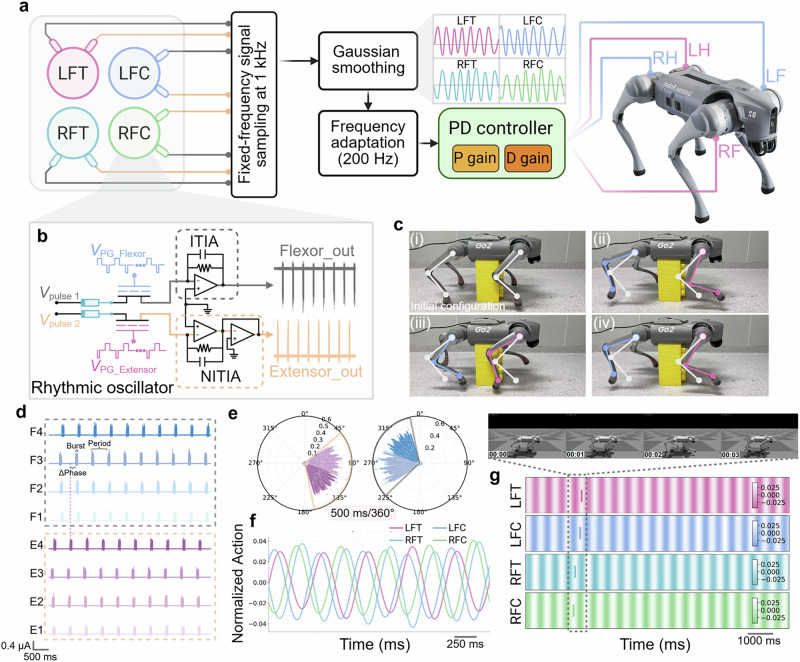
Rhythmic gait control in the quadruped robot. **a** Schematic of distributed 2(PG-TS)-based rhythmic oscillator for locomotion control in Unitree Go2 quadruped robots. **b** Detailed circuit topology and rhythmic spiking outputs of a 2(PG-TS)-based rhythmic oscillator. **c** Physical demonstration of rhythmic quadrupedal limb oscillations in the Unitree Go2 robot. White limb guides indicate initial positions (i). The robot executes a propulsive stride with the RF and LH limbs (pink-colored guides), while retracting the RH and LF limbs (blue-colored guides) (ii). Phase-inverted motion follows, with RH and LF limbs advancing and RF/LH limbs retracting (iii). The quadruped achieves a stable periodic alternating gait (iv). **d** Four-channel rhythmic current spiking outputs (Fi: Flexor_i_; Ei: Extensor_i_; *i *= 1, 2, 3, 4). **e** Phase-modulated rhythmic spiking outputs of flexor-extensor antagonistic pairs. Extensor (red hues) and flexor (blue hues) spikes exhibiting 180° anti-phase synchronization. **f** Rhythmic extensor-flexor spiking signal pairs are Gaussian-shaped into angular gait control signals for PD (proportional-derivative) controllers. **g** Heatmap of sinusoidal gait control signals for angular vectors. Top panel: Rhythmic limb oscillations of the Unitree Go2 quadruped robot in an Isaac Gym simulation, demonstrating stable alternating stepping locomotion.

In contrast to the conventional position-based limb control in quadruped robots, where centralized neural networks allocate target joint positions to PD controllers for torque actuation (Supplementary Fig. 15)^46,47^. We implement rhythmic gait control in the Unitree Go2 via distributed 2(PG-TS) spiking oscillators, establishing a neuromorphic circuit-to-robot control loop (Fig. 4a). As shown in Fig. 4b, fixed-phase-inversion (180°) depolarization/hyperpolarization voltage levels (*V*_PG_Flexor_ and *V*_PG_Extensor_) generate rhythmic spiking outputs. The Flexor_out signal is inverted via an inverted transimpedance amplifier (ITIA) to produce negative-polarity spikes, enabling bidirectional joint angular excursions. Four 2(PG-TS) rhythmic oscillators assigned to left front thigh (LFT), left front calf (LFC), right front thigh (RFT), and right front calf (RFC) joints generate phase-modulated rhythmic spikes, which are converted into sinusoidal joint angle signals through Gaussian smoothing filters. These oscillations drive PD controllers to actuate joint motors, enabling stable quadrupedal locomotion with inherent anti-phase synchronization. Supplementary Video 1 demonstrates stable periodic quadrupedal stepping in the Unitree Go2 robot, achieved via a rhythmic spiking oscillator control scheme based on artificial plateau-like neuronal dynamics. We employ a diagonally synchronized limb grouping strategy (left hind (LH)-right front (RF) and left front (LF)-right hind (RH) pairs). As shown in Fig. 4c, the LH and RF limbs propel forward while the LF and RH limbs retract, with these roles reversing in subsequent cycles under identical rhythmic oscillations, achieving phase-locked quadruped coordination.

Figure 4d displays the raw rhythmic current spiking outputs for quadrupedal gait control in the Unitree Go2 robot. Each extensor-flexor spiking pair shares an identical oscillation period, but with distinct phase distributions: extensor spikes are incrementally distributed from 54° to 162° in 27° steps, while flexor spikes span 234° to 342° with the same step size, enforcing a 180° anti-phase synchronization for biomimetic antagonistic joint control (Fig. 4e)^4^. Supplementary Fig. 16 demonstrates programmable spiking outputs with reduced intensity and narrower phase distributions, highlighting the functional adaptability of the 2(PG-TS) rhythmic oscillators. The four-channel spiking signals are Gaussian-filtered to eliminate high-frequency noise and smoothed for joint angle conversion, generating sinusoidal trajectories for the LFT, LFC, RFT, and RFC joints (Fig. 4f). A grating heatmap (Fig. 4g) visualizes the rhythmic phase relationships and limb coordination, where LFT/LFC signals govern the LF and RH limbs, while RFT/RFC signals drive the RF and LH limbs. The top panel of Fig. 4g shows simulated rhythmic limb oscillations in the Isaac Gym environment, achieving stable alternating gaits (Supplementary Video 2). These results validate the feasibility of programmable plateau neuron-inspired neuromorphic spiking oscillators for quadrupedal rhythmic locomotion. This hardware-to-hardware, end-to-end control paradigm holds the potential to enable autonomous robotic systems.

## On-ground adaptive locomotion of the quadruped robot

**Fig. 5:**
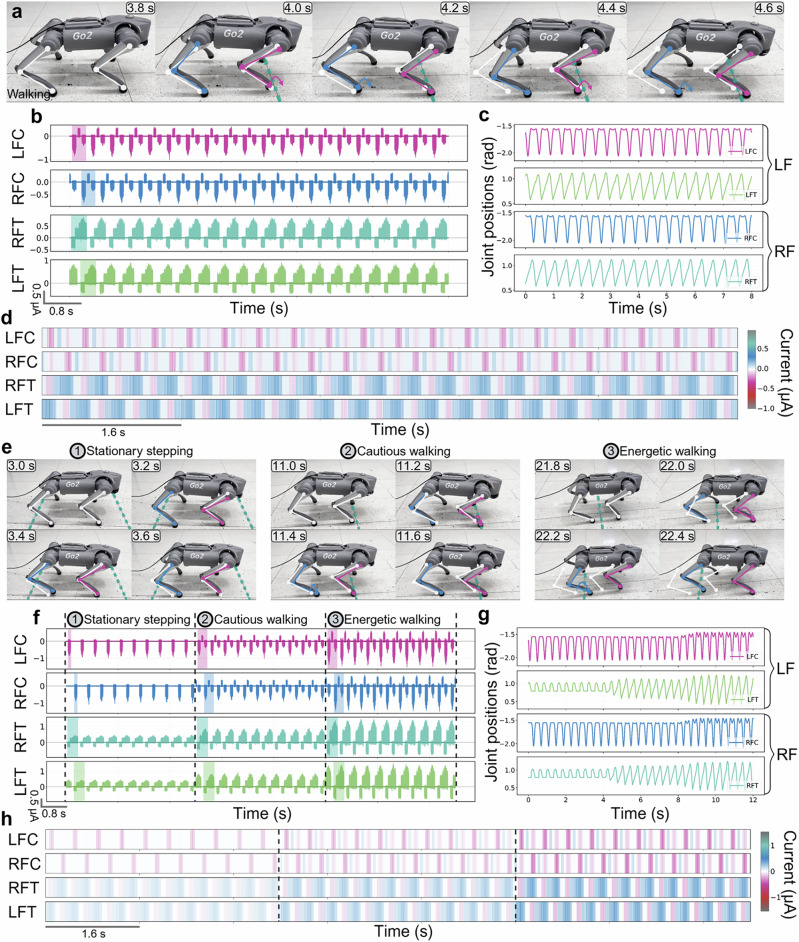
On-ground adaptive locomotion of the quadruped robot. **a** Physical demonstration of the Unitree Go2 robot performing steady on-ground trotting. White limb guides indicate the initial stance. Pink and blue guides highlight the motion of the RF and RH limbs, respectively, under a diagonal synchronization strategy. **b** Rhythmic spike bursts from the four joint actuator groups. **c** Actual joint angle oscillation signals. **d** Heatmap of the rhythmic spike events, illustrating the spatiotemporal pattern for coordinated locomotion. **e** Physical demonstration of adaptive on-ground walking. The adaptive gait sequence consists of three phases: 1. Stationary stepping, 2. Cautious walking, 3. Energetic walking. **f**–**h** Adaptive rhythmic spike bursts modulating across the three phases (**f**), The actual joint angle oscillation curves (**g**), Heatmap of the spike events across the three phases, showing the transition in the spatiotemporal pattern.

We first validate the system’s capability for steady, coordinated trotting. As shown in Fig. 5a, the robot achieved stable forward locomotion at a speed of 0.15 m/s using the diagonal synchronization strategy (Supplementary Video 3). The underlying control signals are presented in Fig. 5b–d: the rhythmic spike bursts generated by the plateau neurons for the LFC, RFC, RFT, and LFT joints (Fig. 5b); the corresponding control voltages are shown in Supplementary Fig. 17, where the left and right limb joints exhibit anti-phase spike output with a 200-ms phase offset. The Gaussian-filtered joint angle trajectories that drove the PD controllers (Fig. 5c), together with a heatmap visualizing the precise spatiotemporal pattern of the spike events that orchestrate the gait (Fig. 5d), complete an integrated data chain that confirms translation from plateau-neuron rhythm generation to effective physical locomotion. We further evaluate the system‑level energy efficiency of this neuromorphic control approach. The joint torque profiles during walking are provided in Supplementary Fig. 18. A comprehensive power analysis (Supplementary Fig. 19) shows that during steady walking at 0.4 m/s, the PG‑TS‑based system reduces the total motor‑drive power by 13.64 % compared to a conventional microcontroller unit (MCU)‑based PD controller.

Beyond steady-state operation, we implement adaptive walking to demonstrate the tunable capability of the plateau neuron hardware. By modulating input voltage to the plateau neuron circuits and enhancing the rhythmic spiking under optical bias (Supplementary Fig. 20), we command the robot to execute a three-phase adaptive sequence: stationary stepping, cautious walking (0.15 m/s), and energetic walking (0.4 m/s) (Fig. 5e) (Supplementary Video 4). The resulting adaptive rhythmic spike bursts, which maintain the 200-ms phase difference between left and right limbs, are shown in Fig. 5f. The smoothly transitioning joint-angle profiles (Fig. 5g) and the evolving spatiotemporal heatmap of spike bursts (Fig. 5h) illustrate the system’s capacity for continuous behavioral modulation based on external inputs. This demonstrates that the in-situ spike malleability of plateau neurons is harnessed for dynamic gait adaptation. Taken together, these experiments provide evidence that our neuromorphic CPG hardware can generate control signals sufficient for effective on-ground locomotion and exhibits the inherent programmability required for adaptive behavior.

## Discussion

We present an artificial plateau neuron (PG-TS) and experimentally validate its bistable control of depolarization and hyperpolarization, along with its in-situ spike malleability, which facilitates rhythmic spike encoding for locomotion control. Based on this device, we implement the bibranched 2(PG-TS) circuit to achieve multimodal control. By manipulating the in-situ spike-malleable action potential, the 2(PG-TS) circuit demonstrates exceptional energy efficiency, approaching that of biological neural systems, presenting a significant advancement over previously reported spike-based motion control circuits. The compact and bioinspired design of the 2(PG-TS) significantly reduces circuit complexity in terms of device count (see quantitative comparison in Table 1). Moreover, both spike frequency and amplitude can be modulated through input excitation and plateau potential, offering enhanced signal encoding flexibility. To further improve rhythmic signal fidelity, we design the 4PG-TS circuit based on the MAC operations. Together, the 2(PG-TS) and 4PG-TS circuit designs provide basic building blocks for developing compact, low-power bioinspired locomotion control systems. Crucially, the pathway to system-level efficiency lies in scalable integration. As conceptualized in Supplementary Fig. 21, these PG-TS cores can be organized into parallel arrays, where the fixed overhead of peripheral circuits is amortized across many cores, ensuring that the intrinsic low energy per spike (~141 pJ/spike) dominates the system’s energy budget for rhythm generation^48,49^. Low-power 2(PG-TS) circuits generate rhythmic extensor/flexor spiking bursts that are Gaussian-filtered into angular oscillation signals. These signals drive PD controllers to achieve joint-level trotting gait control in the Unitree Go2 quadruped robot. The system successfully executes stable on-ground locomotion and exhibits adaptive walking through direct modulation of the plateau neuron’s operating point, confirming its potential for real-world deployment in dynamic environments. This end-to-end gait control framework—enabled by plateau neuron-inspired hardware circuits—could enable hardware-autonomous intelligent robotics. Such rhythm-generating circuits may advance medical applications like neuromorphic prosthetics and motor rehabilitation when integrated with flexible bioelectronic substrates. For practical deployment, a viable path lies in heterogeneous integration, where such neuromorphic cores can be combined with digital silicon CMOS. Furthermore, the artificial plateau neurons with in-situ spike-malleability hold promise for applications in low-power cardiac pacemaker design and emerging SNN in-memory computing architectures.

**Table 1:** Comparison of rhythmic motion control circuits: 2(PG-TS) vs. CMOS ASIC, IMT and organic coupled oscillator

	Biological neural^28^	Satio et al.^50^	Yang et al.^51^	Dutta et al.^10^	Harikesh et al.^28^	Sarkar et al.^26^	This work: 2(PG-TS)
Neuron model	CCVO	Pulse-type	Matsuoka	CO	c-OECN	OAN	Plateau neuron
Bidirectional	Yes	Yes	Yes	Yes	Yes	No	Yes
Transistor count	-	76	1296	4	2	2	2
Frequency	0.5–500 Hz	2 Hz	0.5–2 Hz	12 Hz	100 Hz	6–40 Hz	0–500 Hz
Energy/spike	100 pJ/spike	69 µJ/spike	30 µJ/spike	2.67 µJ/spike	175 nJ/spike	57 nJ/spike	141.37 pJ/spike
Power	50 nW	138 µW	60 µW	32 µW	17.5 µW	143 µW	141.37 nW

*IMT* insulator-to-metal phase transition, *CMOS* complementary metal oxide semiconductor, *ASIC* application-specific integrated circuit, *CCVO* current-controlled voltage oscillation, *OAN* organic artificial neurons, *c-OECN* conductance-based Organic electrochemical neurons.

## Methods

### MoS_2_ CVD synthesis

A silicon (Si) chip was prepared by immersing it in an aqueous solution of sodium molybdate (Na_2_MoO_4_) with a concentration of 12 mg/mL, followed by air drying. Subsequently, this Si chip and a zinc sulfide (ZnS) crystal plate were sequentially positioned above a sapphire (Al_2_O_3_) substrate, using mica spacers between each layer, and collectively inserted into a chemical vapor deposition (CVD) furnace. The furnace chamber was purged with Ar gas (300 sccm) and heated to the designated growth temperature of ~800 °C. Throughout the growth period, the growth duration was controlled between 30 to 60 min. After the completion of the growth process, the system was allowed to cool down naturally to room temperature.

### MoS_2_ film transfer

Initially, a PMMA layer was spin-coated onto MoS_2_/sapphire at ~1500 rpm for 1 min and subsequently baked in air at 120 °C for 5 min. Following this, a TRT piece, pre-punched with a hole, was attached to the PMMA/MoS_2_/sapphire assembly. Then the TRT/PMMA/MoS_2_ composite was detached from the sapphire in water and allowed to dry in air before being laminated onto a 2-inch SiO_2_/Si substrate. The TRT was removed by heating to the designated release temperature and then peeling off. To further improve the interaction between MoS_2_ and the SiO_2_/Si, the PMMA/MoS_2_/SiO_2_/Si assembly was baked at 180 °C for 10 min. It was then washed with acetone and annealed in Ar (300 sccm) at 400 °C for 4 h to remove the PMMA, leaving MoS_2_ on SiO_2_/Si.

### Graphene CVD synthesis

A single crystal Cu(111) alloy foil (25 μm thick, Zhongke Crystal Materials Co. Ltd) was initially placed on a quartz substrate and introduced into the chemical vapor deposition (CVD) furnace. The system was then heated to 1100 °C under a reducing atmosphere of 500 standard cubic centimeters per minute (sccm) argon (Ar) and 30 sccm hydrogen (H_2_). Following this, a gas mixture of 0.01 sccm methane (CH_4_), 10 sccm H_2_, and 500 sccm Ar was introduced for 60 min to obtain monolayer graphene.

### Graphene film transfer

The graphene film was transferred to a 2-inch wafer using a standard wet transfer method. An 8% polymethyl methacrylate (PMMA) solution in anisole was spin-coated onto the as-grown graphene/Cu, followed by baking at 120 °C for 2 min. The Cu substrate was then etched using a 0.2 mol/L ammonium persulfate ((NH_4_)_2_S_2_O_8_) solution. The resulting PMMA/graphene film was gently placed onto the SiO_2_/Si substrate, followed by a 10-min bake at 120 °C. Finally, the PMMA/graphene/bottom structure was annealed in an Ar/H_2_ atmosphere at 400 °C for 10 h to completely remove the PMMA layer.

### 2-inch wafer PG-TS, 2(PG-TS), and 4PG-TS fabrication

The fabrication process commenced with a 2-inch p-doped silicon wafer featuring a 285 nm SiO_2_ layer. The MoS_2_ channel regions were subsequently patterned using Direct Write Lithography (DWL), followed by Inductively Coupled Plasma (ICP) etching with O_2_/Ar to remove the exposed areas. Next, the source and bottom of the TS electrodes were patterned by DWL, Au (30nm) deposited via Electron-Beam Evaporation (EBE), followed by a lift-off process. Post the lift-off process, a 1 nm Al seed layer was coated by EBE. This was followed by a 5-min annealing step at 95 °C in ambient air. The tunneling layer Al_2_O_3_ (7 nm) was deposited on the bottom electrodes using an Atomic Layer Deposition (ALD) system. Afterward, 2-in wafer-scale CVD Graphene was transferred onto the Al_2_O_3_ and dry-etched by ICP to form a floating trap. Ag/Au (20/30 nm) top of TS deposited by EBE for pulses input. Then, a 1 nm Al seed layer coated by EBE, the blocking layer Al_2_O_3_ (25 nm) was deposited by ALD on the seed layer. Finally, the gate (Au 30 nm) of *V*_PG_ electrodes was coated by EBE. The fabrication was completed with 2-inch PG-TS, 2(PG-TS), and 4PG-TS units. These units subsequently underwent a 2-h annealing treatment at 300 °C under H_2_/Ar (20/150 sccm), ~1 Torr.

### Electrical characterization

The electrical measurements were performed under ambient atmospheric conditions at room temperature using a probe station equipped with a Keithley 4200 Semiconductor Characterization System. For the DC measurements, the 4200-SMU source-measure unit was utilized in DC mode. Meanwhile, the 4200-PMU waveform generator/pulse measure unit was employed for the pulsed mode measurements. The use of the industry-standard Keithley 4200 platform ensures the integrity and reliability of the electrical characterization data presented in this work.

### Quadruped robot actuations

We employed the Unitree Go2 quadruped robot as a simulation and physical experimental platform to validate the efficacy of rhythmic spiking signals, generated by artificial plateau neuronal circuits, in real-world motor control applications. The Unitree Go2 platform comprises four legs, each with three joint actuators (hip, thigh, and calf), totaling 12 motors. During experiments, all hip joints were immobilized, leaving eight actively controlled motors (thigh and calf joints per leg). For these 8 joints, each motor’s initial angle was set to ${{{{\boldsymbol{\theta }}}}}_{{{\rm{init}}},{{\rm{i}}}}={\left[{\theta }_{{{\rm{init}}},1},{\theta }_{{{\rm{init}}},2},\cdots {\theta }_{{{\rm{init}}},8}\right]}^{{{\rm{T}}}}$, while the neuromorphic circuit generated a relative angular displacement $\Delta {{{{\boldsymbol{\theta }}}}}_{{{\rm{i}}}}={\left[\Delta {\theta }_{1},{\Delta \theta }_{2},\cdots {\Delta \theta }_{8}\right]}^{{{\rm{T}}}}$. The final motor command was computed as:

2 $${{{{\boldsymbol{\theta }}}}}_{{{\rm{i}}}}={{{{\boldsymbol{\theta }}}}}_{{{\rm{init}}},{{\rm{i}}}}+\Delta {{{{\boldsymbol{\theta }}}}}_{{{\rm{i}}}}$$

To derive $\Delta {{{{\boldsymbol{\theta }}}}}_{{{\rm{i}}}}={\left[\Delta {\theta }_{1},{\Delta \theta }_{2},\cdots {\Delta \theta }_{8}\right]}^{{{\rm{T}}}}$, we subtracted antagonistic spiking signal pairs (Ei, Fi) from 2(PG-TS) rhythmic oscillators. The differential signals were then Gaussian-filtered to enhance waveform smoothness and suppress transient noise.

For locomotion coordination, we implemented a diagonal synchronization strategy: the LF and RH limbs shared identical motion profiles, while the RF and LH limbs mirrored this synchronization. This approach enabled the use of four (Ei, Fi) signal pairs to control all 8 active joints, achieving decentralized, energy-efficient interlimb coordination without centralized computational overhead.

## Supplementary information

Supplementary Information

Peer Review file

Description of Additional Supplementary Files

Supplementary Movie 1

Supplementary Movie 2

Supplementary Movie 3

Supplementary Movie 4

## Data Availability

The data supporting the quadruped robot application findings in this study are openly available in the Figshare database at 10.6084/m9.figshare.31746325. Detailed research data supporting the plots of this study are available from the corresponding authors upon request.

## Code availability

The code supporting the configuration of the robot in this study is available at https://github.com/chaiqingao/go2_control with unrestricted access.
