# Supplementary material for: Artificial plateau neurons with in-situ spike-malleability for rhythmic quadrupedal locomotion
Source: Nat Commun. 2026 Apr 28;17:5801. doi: 10.1038/s41467-026-72428-2 (PMC13332192; doi:10.1038/s41467-026-72428-2)
Supplement: Supplementary file 1 — Supplementary Information [file 41467_2026_72428_MOESM1_ESM.pdf]

Supplementary Information

Artificial Plateau Neurons with *In-situ* Spike-Malleability for Rhythmic Quadrupedal Locomotion

Hailiang Wang[1], Yishu Zhang[1,2]*, Qingao Chai[3,4], Qian He[1], Jiayang Hu[1], Yongqing Bai[1], Zongwen Li[1], Jian Chai[1], Xin He[2], Yu Fu[6], Mengze Zhao[5], Guodong Xue[5], Kaihui Liu[5], Huajin Tang[3,4]*, Yang Xu[1]* and Bin Yu [1]*

[1] College of Integrated Circuits, Zhejiang University, Hangzhou, Zhejiang 311200, China.
[2]ZJU-Hangzhou Global Scientific and Technological Innovation Center, Hangzhou, Zhejiang 310020, China.
[3]College of Computer Science and Technology, Zhejiang University, Hangzhou, 310027, China.
[4]State Key Lab of Brain-Machine Intelligence, Zhejiang University, Hangzhou, 310058, China.
[5]State Key Laboratory for Mesoscopic Physics, Frontiers Science Center for Nano-Optoelectronics, School of Physics, Peking University, Beijing 100871, China.
[6]Key Laboratory of Quantum State Construction and Manipulation, Department of Physics, Renmin University of China, Beijing 100872, China.
*Email: zhangyishu@zju.edu.cn; htang@zju.edu.cn; yangxu-isee@zju.edu.cn; yu-bin@zju.edu.cn (Lead Contact)

Table of contents

Supplementary Note 1. Power dissipation and energy consumption of 2(PG-TS) structure.

Supplementary Figure 1. Schematic illustration of fabrication process.

Supplementary Figure 2. Raman spectroscopy characterization.

Supplementary Figure 3. AFM topography data of single-layer MoS_2 thin film.

Supplementary Figure 4. AFM topography data of single-layer Graphene thin film.

Supplementary Figure 5. Electrical characteristics of the Al_2O_3-TS.

Supplementary Figure 6. The IF TS with stimuli-modulated output spike fir-rate.

Supplementary Figure 7. The energy band diagram schematic of the stacked PG.

Supplementary Figure 8. Electrical characteristics of the PG elements.

Supplementary Figure 9. SEM images of PG-TS structure.

Supplementary Figure 10. The continuous voltage pulse stimulation (V_{Pulse}) applied to drain.

Supplementary Figure 11. The depolarization and hyperpolarization of the PG-TS device.

Supplementary Figure 12. Switching dynamics of depolarized PG-TS (experiments).

Supplementary Figure 13. The schematic of coupled Bio-CPG unit and 2(PG-TS) circuit.

Supplementary Figure 14. The spikes corresponding to successive depolarization.

Supplementary Figure 15. Common position-based method.

Supplementary Figure 16. Amplitude and phase programmability of Rhythmic Spikes.

Supplementary Figure 17. Control voltage waveform for rhythmic spike output.

Supplementary Figure 18. Measured joint torque during energetic walking of Unitree Go2 robot.

Supplementary Figure 19. System-level power analysis of quadrupedal walking.

Supplementary Figure 20. Control voltages waveform for the three-phase locomotion.

Supplementary Figure 21. Conceptual architecture for a scalable neuromorphic locomotion system.

Supplementary Table 1. Comparison of activation latency and related performance metrics for robotic control

Supplementary Note 1. Power dissipation and energy consumption of 2(PG-TS) structure.

The instantaneous power and energy consumption calculations are based on the fundamental operating state of 2(PG-TS) circuit: PG depolarization strength V_{PG} = -10 V, 10 ms; V_{Pulse} = 0.6 V, T_{pulse} = 1 ms, duty cycle = 50%. The instantaneous power is calculated as $P_{inst} = V_{pulse}I_{spike}$, where V_{pulse} and I_{spike} represent the supply voltage and spike current of 2(PG-TS) circuit, respectively. The calculations include 1200 spikes shown in Fig. 4e, with an average peak current I_{mean} = 0.23562 μA and an average power dissipation of P_{mean} = 141.37 nW. The average current of the 1000 non-spike samples is $I_{non\text{-}spike}$ = 0.01382 μA << I_{mean}, so the quiescent power dissipation is negligible in energy consumption calculation. Using $E_{spike} = P_{mean}T_{pulse}$, the energy consumption per spike is determined to be E_{spike} = 141.37 pJ/spike. Furthermore, a weaker PG depolarization V_{PG} = -8 V, 10 ms (as in Coding 3), E_{spike} can be reduced further to as low as 91.968 pJ/spike in 1000 spikes.

Supplementary Figures

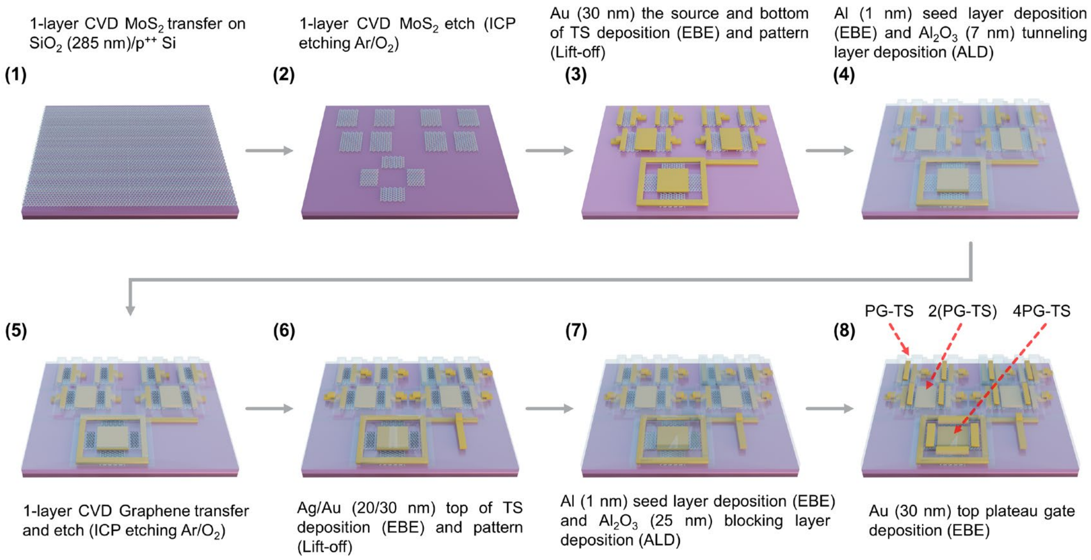

Supplementary Figure 1. Schematic illustration of fabrication process. (1) 1-layer CVD MoS_2 transferred on SiO_2 (285 nm)/p^{++} Si. (2) 1-layer CVD MoS_2 etched for channel (ICP etching Ar/O_2). (3) The source and bottom of TS electrodes patterned by DWL, Au (30 nm) deposited via EBE, followed by a lift-off process. (4) A 1 nm Al seed layer coated by EBE. The tunneling layer Al_2O_3 (7 nm) deposited on the bottom electrodes using an ALD. (5) 1-layer CVD Graphene transferred etched for floating layer (ICP etching Ar/O_2). (6) The top of TS electrodes patterned, and Ag/Au (20/30 nm) deposited by EBE, followed by a lift-off process. (7) A 1 nm Al seed layer coated by EBE. The blocking layer Al_2O_3 (25 nm) deposited by ALD. (8) The gate (Au 30 nm) of PV electrodes coated by EBE.

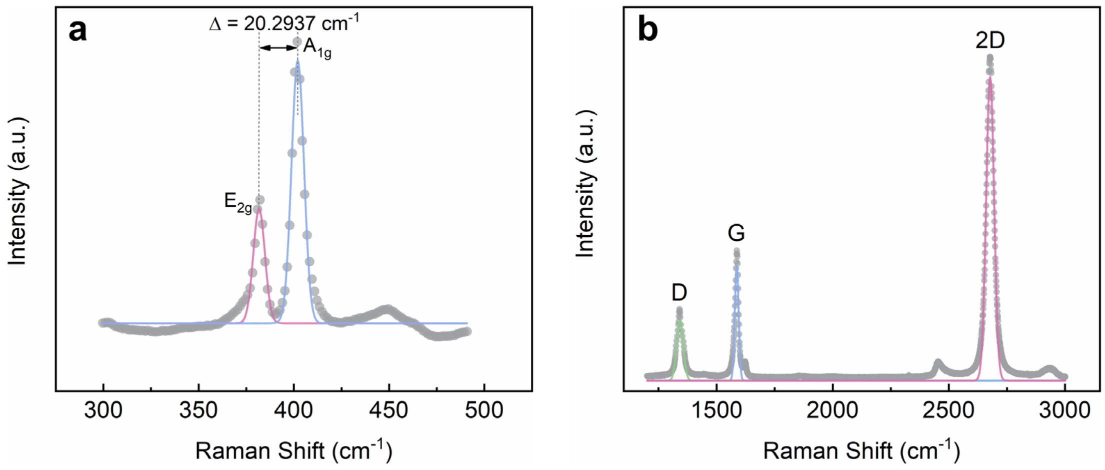

Supplementary Figure 2. Raman spectroscopy characterization. a, Raman spectroscopy shows the E^1_{2g} and A_{1g} peaks and Δ between peaks, which indicates single layers of MoS_2. **b,** The Raman spectrum of the graphene exhibits the characteristic D, G, and 2D peaks. The amplitude of the 2D and G peaks indicates the graphene is monolayer. The presence of the D peak and the small peak at the edge of the G peak suggest the graphene contains a certain degree of defects, which can be utilized for electron trapping

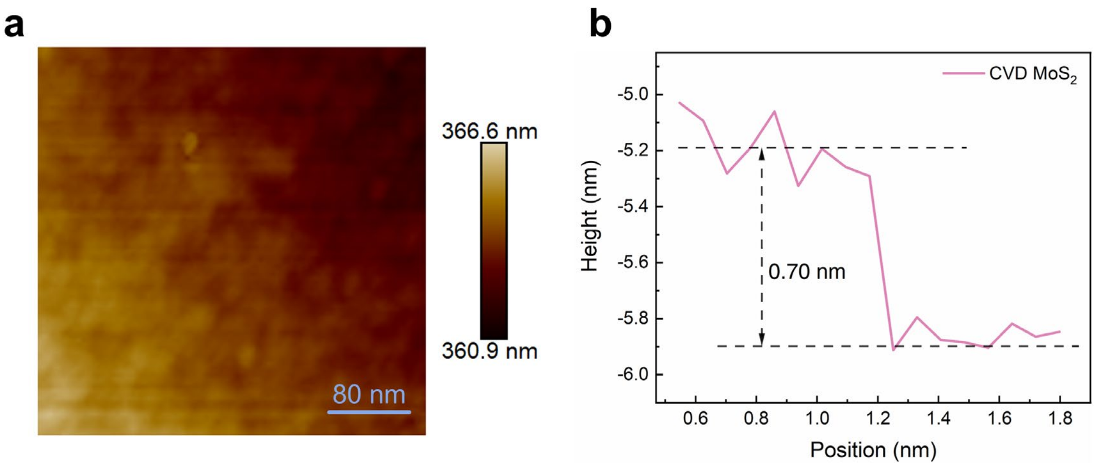

Supplementary Figure 3. AFM topography data of single-layer MoS_2 thin film. a, AFM height image of the single layer CVD MoS_2 on the substrate, which displays the uniformity of the materials. **b,** Testing thin film thickness using AFM step method, identified as 0.70 nm.

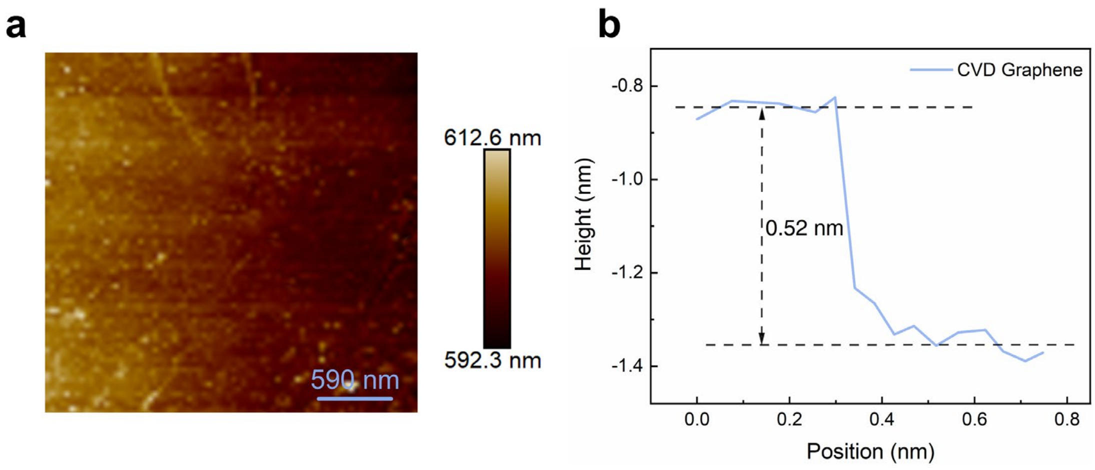

Supplementary Figure 4. AFM topography data of single-layer Graphene thin film. a, AFM height image of the single layer CVD Graphene on the tunneling layer. **b,** Testing thin film thickness using AFM step method, identified as 0.52 nm.

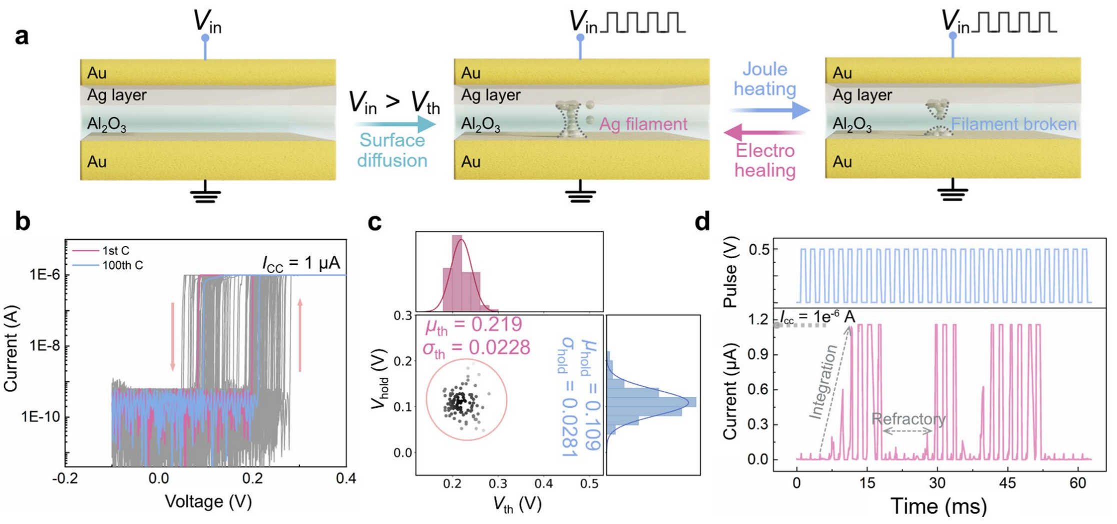

Supplementary Figure 5. Electrical characteristics of the Al_2O_3-TS. a, Illustration of the lifecycle of Ag filaments within the Al_2O_3-TS structural units. V_{th}, threshold voltage for filament initiation. The transient resistive switching behavior observed in TS is attributed to the formation and rupture of Ag conductive filaments (CFs) under appropriate driving voltages. **b**, Typical volatile threshold resistive switching of the Ag/Al_2O_3/Au cell in 100 cycles, exhibiting steep switching slope. I_{cc}, compliance current. **c**, Statistical analysis of the V_{th}, and V_{hold} observed 100 cycling sweep processes ($\mu_{th} = 0.219$, $\sigma_{th} = 0.02s28$, $\mu_{hold} = 0.109$, and $\sigma_{hold} = 0.0281$). The tightly-clustered values within a centrally symmetric range indicate robust and consistent switching characteristics of TS component, providing a crucial foundation for stable, rhythmic spike modulation. **d**, Showcasing the current-integrating and threshold-triggered spiking dynamics of the Al_2O_3-TS structural units under persistent pulse stimulation ($V_{Pulse} = 0.5$ V, $T_{interval} = 1$ ms, duty-cycle = 50%).

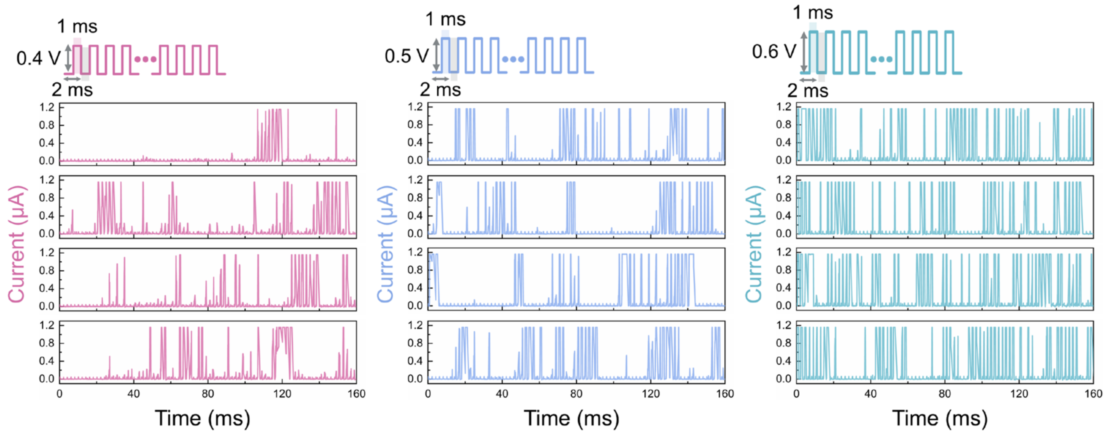

Supplementary Figure 6. The integrate-and-fire TS with stimuli-modulated output spike fir-rate. The raster plot of fir-rate versus time for the neuron transistor under various pulse amplitudes of 0.4 V, 0.5 V, and 0.6 V, respectively (time interval = 1 ms, width = 1 ms). The spiking rate of the TS neuron component is observed to increase significantly with the enhancement of the input continuous pulse amplitude, indicating that the neuronal activity of the TS can be modulated by the strength of the input stimuli.

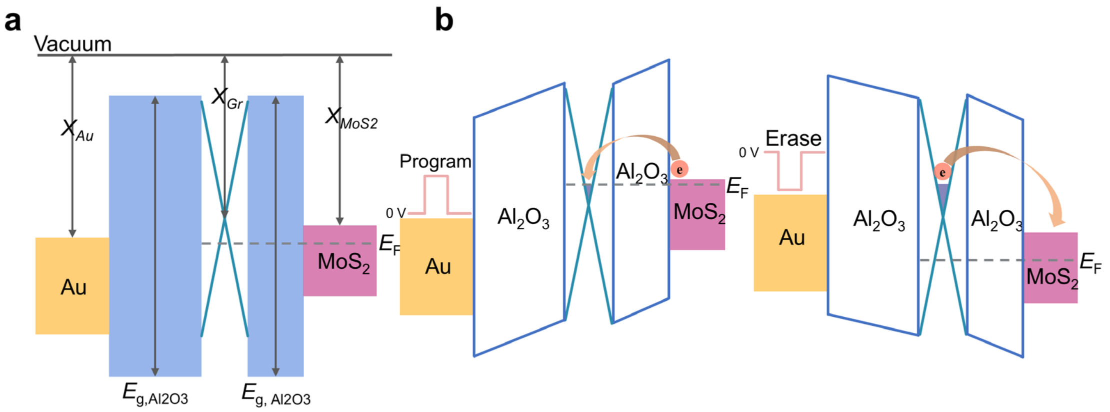

Supplementary Figure 7. The energy band diagram schematic of the stacked PG. a, The flat band state of the heterostructure memory, where X is the electron affinity, E_F is the Fermi level and E_g is the bandgap. **b,** e- trapped in floating layer (Graphene); negative voltage applied to erase e- in Graphene.

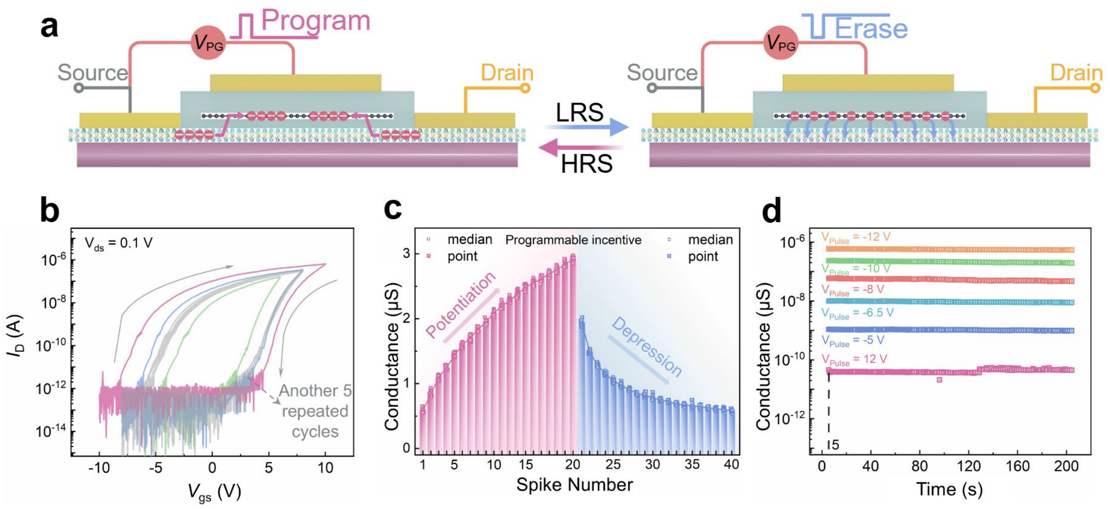

Supplementary Figure 8. Electrical characteristics of the PG elements. a, Visualizing program and erase-based configuration switching in the PG structural unit. **b**, The PG structural unit transfer curves with exceptionally expansive switching ratio, which exhibits pronounced hysteresis window. The gray trace represents the result from 5 repeated seep scans. V_{gs}, gate sweep voltage. **c**, The PG structural unit conductance exhibits gradual strengthening and weakening. This Programmable incentive represents one of the critical characteristics for realizing the plasticity of plateau neuronal spiking. Each programmed state includes 20 repeated data points. **d**, The representative PG structural unit conductance distribution exhibit distinct programmed states. These programmed states demonstrate non-volatile behavior, which is central to emulating the memory plasticity observed in biological plateau-neuron spike dynamics.

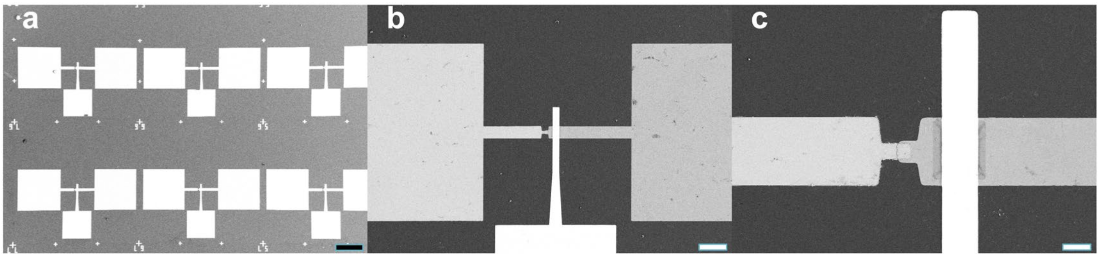

Supplementary Figure 9. SEM images of PG-TS structure. Local areas with different magnifications of (a) 44 X, (b) 188 X, and (c) 1.04 kX. The images indicate that the length of the PG channel is 10 μm and TS connected in series to the drain is 4 μm × 4 μm.

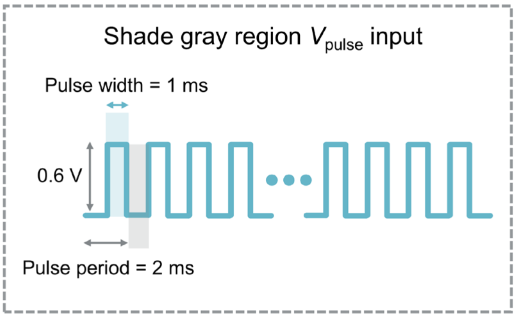

Supplementary Figure 10. The continuous voltage pulse stimulation (V_{Pulse}) applied to drain. In this study, V_{Pulse} applied to the PG-TS, 2(PG-TS), and 4PG-TS structures all utilized the following parameters: V_{Pulse} = 0.6 V, $T_{interval}$ = 1 ms, duty-cycle = 50%. The use of this standardized V_{Pulse} stimulation highlights the generality of PG-TS and its scalable circuit topologies.

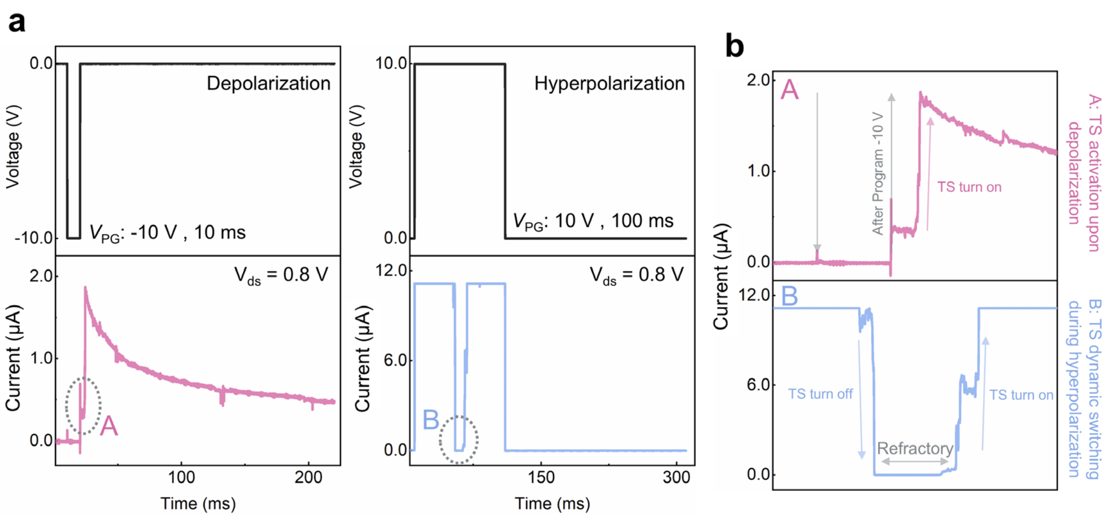

Supplementary Figure 11. The depolarization and hyperpolarization of the PG-TS device. a, The PG-TS device undergoes a depolarization voltage operation (-10 V, 10 ms) that maintains it in an active state, as manifested by the nonlinear self-decay dynamics of its high conductance. After a hyperpolarization voltage operation (10 V, 100 ms), PG-TS returns to a fully quiescent state (V_{read} = 0.1 V). **b**, (A) Following the programming operation, volatile TS component exhibits a transient current spike, indicating a dynamic impedance matching behavior within the PG-TS activation sequence. (B) During the erase process, the probabilistic volatile switching characteristics of TS generate the fire interval.

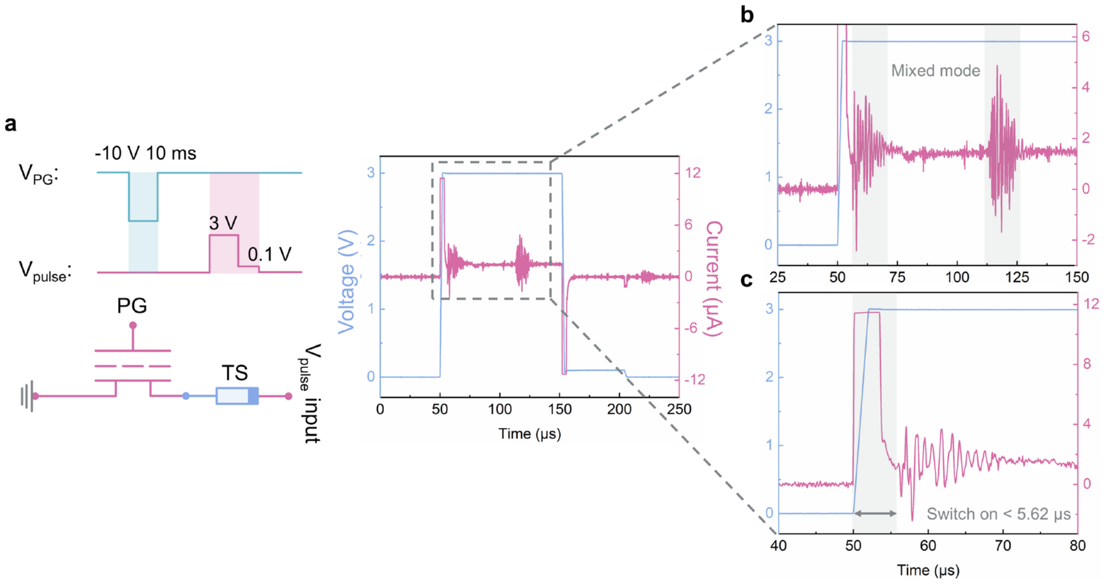

Supplementary Figure 12. Switching dynamics of depolarized PG-TS (experiments). a, V_{PG} (-10 V, 10 ms) bias depolarizes the PG-TS, transitioning it to an active state. A large-amplitude V_{Pulse} (3 V, 100 μs) then triggers spiking output, with a 50 μs V_{read} demonstrating the spiking-off. **b**, Under large-amplitude pulse stimulation in the depolarized state, the PG-TS generates high-amplitude spikes intermixed with self-oscillatory neuromorphic behavior, exhibiting complex mixed-mode dynamics. This intermittent oscillation may be attributed to Joule heating effects, which enhance the volatile[1]. **c**, The dynamic response of spiking activation in the depolarized PG-TS is limited by test precision, with a speed of less than 5.62 μs.

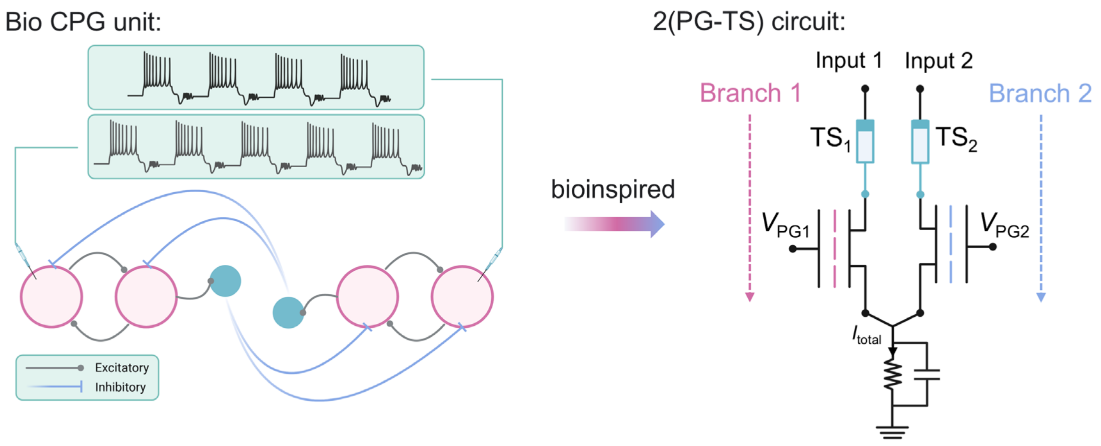

Supplementary Figure 13. The schematic of coupled Bio-CPG unit and 2(PG-TS) circuit. The conceptual illustration of the coupled CPG unit in biological systems, which output complementary muscle actuation signals, involves the participation of various modality-specific neuronal populations in the excitation-inhibition process. At the core, this represents a highly integrated rhythmic signal generator[2]. Inspired by this biological structure, the 2(PG-TS) circuit design we develop also aims to serve as a highly integrated rhythmic signal generator. The two branches (Branch 1 and Branch 2) of this circuit architecture respectively output phase-offset rhythmic control signals to emulate extensor and flexor actuation.

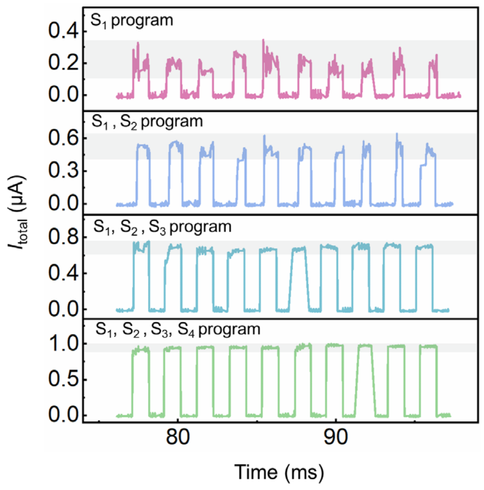

Supplementary Figure 14. The spikes corresponding to successive depolarization. The stepwise depolarization of the four parallel PGs extracts spikes that show the expanded dynamic range enabled by the MAC operation. With the accumulation of parallel depolarization strength, spikes phase stability is progressively enhanced (spike jitter amplitude/spike amplitude), generating more stable rhythmic control signals.

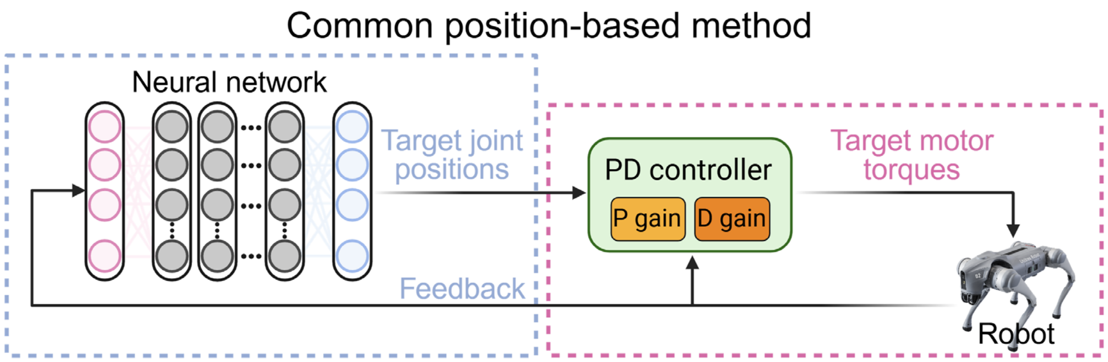

Supplementary Figure 15. Common position-based method. Position-based control paradigm with dual modules: A neural network (blue) generates target joint positions for PD controllers to actuate torque-driven limb motion (pink), while proprioceptive feedback from quadrupedal joints is dynamically corrected via a reinforcement learning (RL)-network[3].

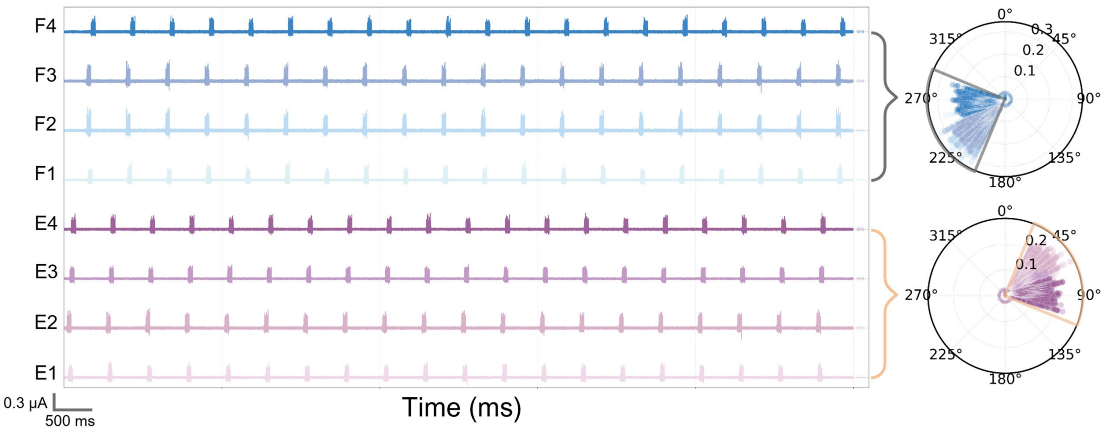

Supplementary Figure 16. Amplitude and Phase Programmability of Rhythmic Spikes. Reduced-amplitude rhythmic spiking outputs across four channels (Fi: Flexori; Ei: Extensori; i = 1, 2, 3, 4) with refined phase distributions. Extensor spikes incrementally distribute from 21.6° to 108° in 14.5° steps, while flexor spikes span 201.6° to 288° at identical step resolution, enforcing 180° anti-phase synchronization for biomimetic antagonistic joint control.

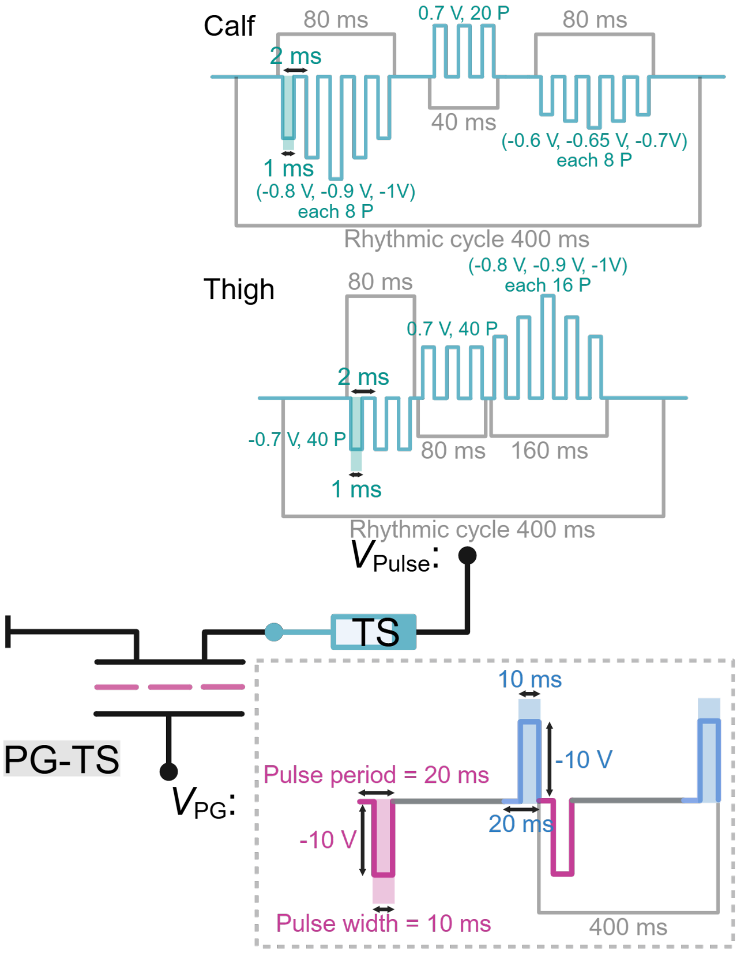

Supplementary Figure 17. Control voltages waveform for rhythmic spike output. The rhythmic period is 400 ms. Within each period, V_{PG} is a periodic depolarizing/hyperpolarizing voltage with 50% duty cycle and an amplitude of 10 V. V_{Pulse} (50% duty cycle and a period of 2 ms) for the calf joints, V_{Pulse} exhibits a staircase voltage that rises and then falls between 60–140 ms and 260–340 ms, and a constant 0.7 V input between 180–220 ms. For the thigh joints, V_{Pulse} provides a constant –0.7 V input between 40–120 ms, a constant 0.7 V input between 120–200 ms, and a staircase voltage that rises and then falls between 200–400 ms.

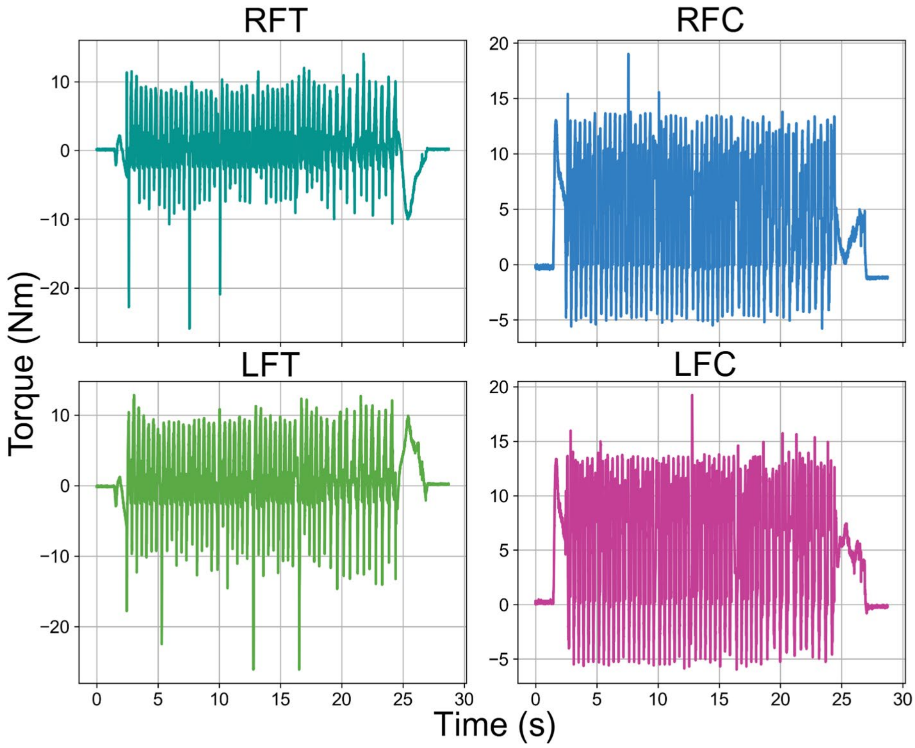

Supplementary Figure 18. Measured joint torque during energetic walking of the Unitree Go2 robot. The plot records the torque outputs from the four key actuation joint groups—RFT, RFC, LFT, and LFC—during a stable walking phase at 0.4 m/s. The data indicate that the calf joints generate larger torque amplitudes to propel the faster forward stepping characteristic of the energetic gait.

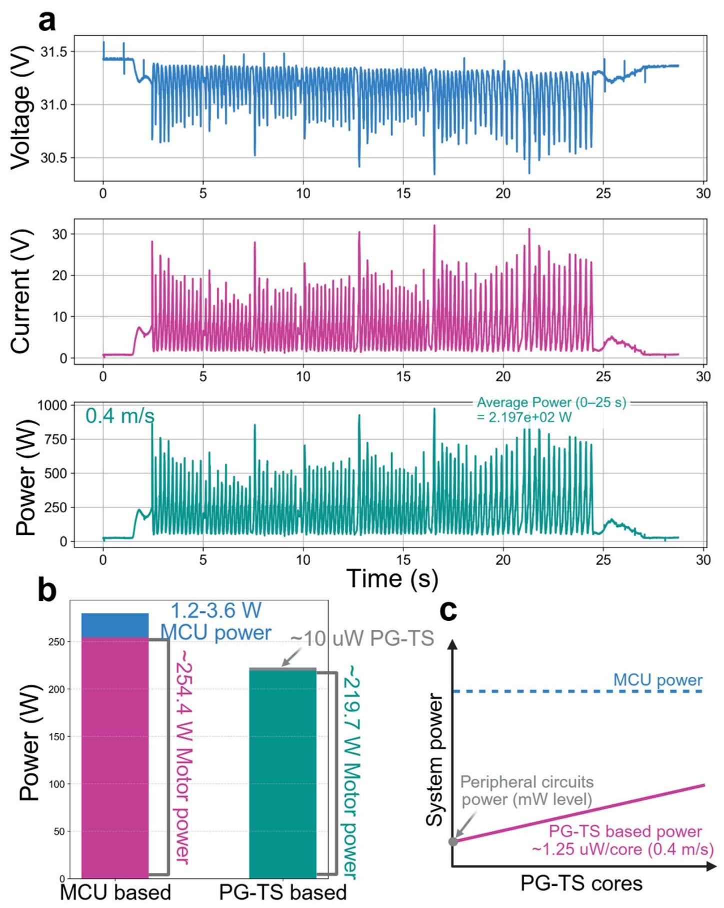

Supplementary Figure 19. System-level power analysis of quadrupedal walking. a, The real-time total power consumption of all joint motors on the Unitree Go2 robot during energetic walking, calculated from the recorded driving voltages and currents. The average power over the 0-25 s period is 219.7 W. b, A direct comparison of the average motor-drive power under two control paradigms for the same robot and gait condition. Conventional MCU-based software control consumes approximately 254.4 W (in the absence of algorithmic power optimization). In contrast, the PG-TS-based neuromorphic hardware control reduces the power to 219.7 W, representing a 13.64% decrease. The PG-TS circuits operate at an ultra-low power: under full load (V_{Pulse} = 1.0 V), the average spiking current is 1.24688 µA, leading to a per-core power of ~1.25 µW. The total estimated power for the eight PG-TS cores driving all joints is thus ~10 µW (not yet including fixed peripheral overhead). c, Scaling analysis of the system power. While the total motor power is dominant, the neuromorphic control circuitry power scales favorably. As conceptualized in Supplementary Fig. 19, in a scaled system with many PG-TS cores sharing fixed peripheral circuits, the total control system power increases approximately linearly with the number of cores. This contrasts with digital architecture where frequent data movement and computation incur high dynamic power, highlighting the efficiency potential of the in-memory, analog rhythmic generation paradigm[4].

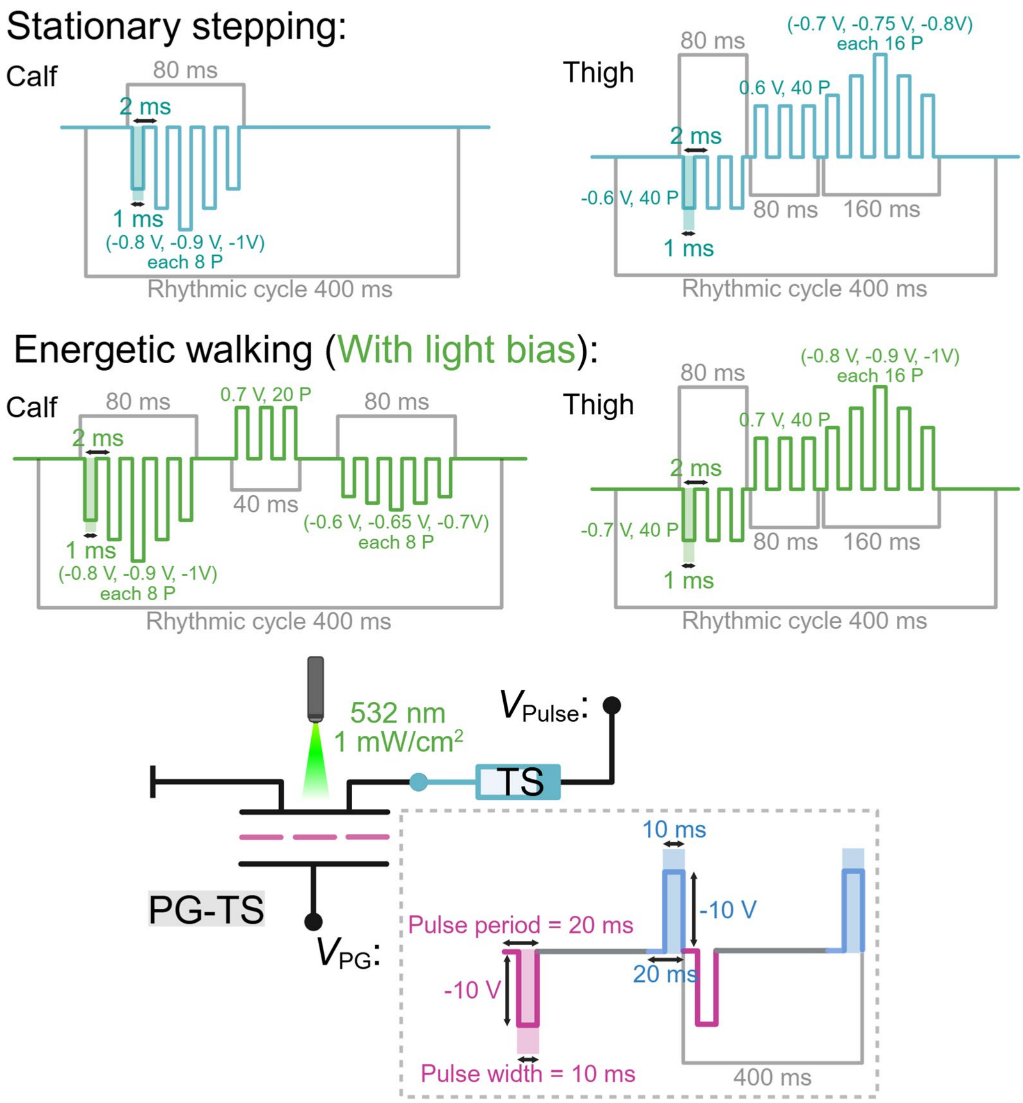

Supplementary Figure 20. Control voltages waveform for the three-phase locomotion. V_{PG} is a periodic depolarizing/hyperpolarizing voltage with 50% duty cycle and an amplitude of 10 V. Compared to steady slow walking, the stationary-stepping phase lacks the reverse-oscillation input stage and the small-amplitude staircase input stage for calf. Leveraging 2D optical response of the plateau neurons, a visible-light (532 nm) bias of 1 mW/cm^2 applied under the pulse conditions for slow walking. The rhythmic spikes output exhibit photo-adaptive enhancement, thereby meeting the large-amplitude swing required for the robot's energetic walking.

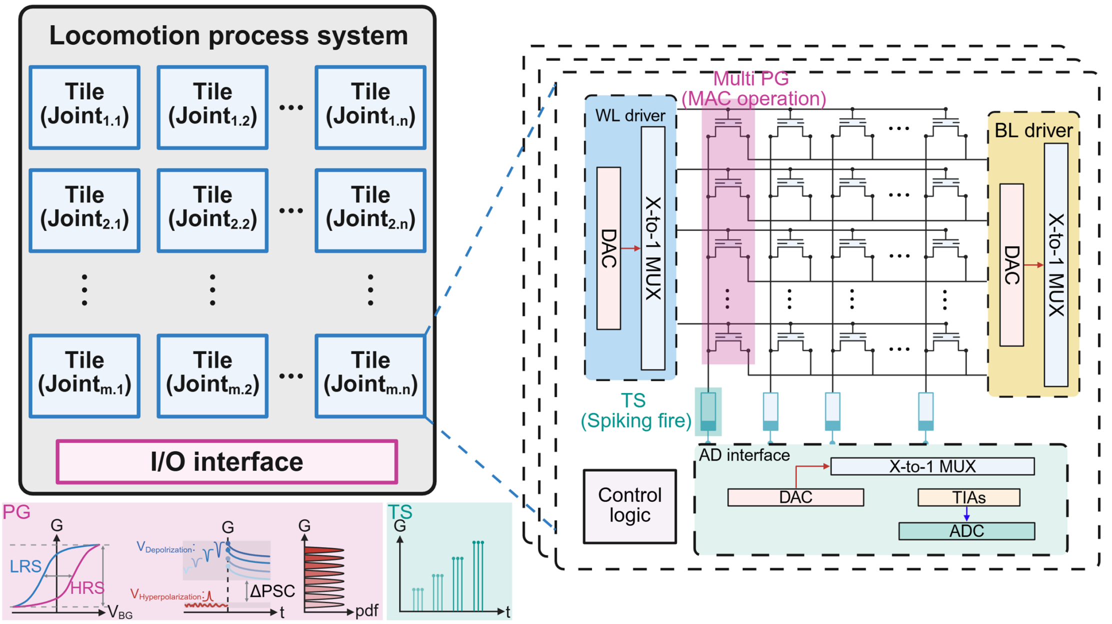

Supplementary Figure 21. Conceptual architecture for a scalable neuromorphic locomotion system. The overall locomotion control system comprises multiple independent Tiles, each responsible for controlling a specific set of robotic joints. Each Tile integrates the PG array with parallel TS output channels. It also incorporates common peripheral circuits essential for system function, including inverting control circuits, TIA, analog-to-digital/digital-to-analog converters (ADC/DAC), and multiplexers (MUX). c, Building upon the experimentally validated 4PG-TS circuit (lower left), the PG array can be scaled to a larger configuration. In such an array, the output rhythmic spiking intensity can be precisely modulated through MAC operations and linear regression, enabled by programming the weight of each PG via its V_{PG} voltage. This scalable, multi-channel architecture is particularly suited for controlling complex joint assemblies (e.g., finger joints), where a single macro-joint is driven by multiple micro-joints. The parallel outputs of a (Multi PG)-TS Tile can generate synchronized rhythmic spikes to coordinate the motion of all sub-joints simultaneously.

Supplementary Table 1 Comparison of activation latency and related performance metrics for robotic control

	**Hashemkhani et al.[5]**	**Bonagiri et al.[6]**	**Yang et al.[7]**	**Chen et al.[8]**	**Dutta et al.[9]**	**This work**
Architecture	Hierarchical SNN with DSM	VO_2 IMT-O	NbO_2 SCNC	Mixed tactile SNN	VO_2 IMT-NO	2(PG-TS)
Activation Latency	~10 ms	~ms scale	~100 µs	~1.2 ms	~83 ms	~5.62 µs
Neuronal Dynamics	Rhythmic spike bursts	Relaxation oscillation	Mixed spiking/bursting	LIF spiking	Relaxation oscillation	Rhythmic spike bursts
Modulation	Phase	Phase	Spiking-bursting transition	Feature extraction	Phase	Amplitude, phase, frequency
Energy per Spike	68 mJ/event	-	1.06 pJ (theor.)	72 nJ	2.67 µJ	141.37 pJ
Process Technology Implementation	Software (Raspberry Pi/Arduino)	Hybrid (VO_2 + CMOS model)	Hybrid (NbO_2 + R/C on PCB)	Hybrid (TENG + CMOS)	Hybrid (VO_2 + CMOS)	Wafer-scale 2D heterostructure
Application Demonstrated	Quadruped obstacle avoidance	Six quadruped gaits (sim.)	Robotic obstacle avoidance	Object classification	Quadruped gaits (sim.)	Quadruped trotting (sim. & robot)

SNN Spiking neural network, DSM dynamic state machine, IMT insulator-to-metal phase transition, NO nano oscillators, SCNC sensorimotor control neural circuit, CMOS complementary metal oxide semiconductor, R/C Resistance/Capacitance, TENG triboelectric nanogenerator.

References

1 Milano, G. *et al.* Quantum Conductance in Memristive Devices: Fundamentals, Developments, and Applications. *Adv Mater* **34**, e2201248 (2022).

2 Grillner, S. & El Manira, A. Current Principles of Motor Control, with Special Reference to Vertebrate Locomotion. *Physiol Rev* **100**, 271-32 (2020).

3 Chen, S., Zhang, B., Mueller, M. W., Rai, A. & Sreenath, K. in *2023 IEEE-RAS 22nd International Conference on Humanoid Robots (Humanoids).* 1-8 (2023).

4 Kudithipudi, D. *et al.* Neuromorphic computing at scale. *Nature* **637**, 801-812, doi:10.1038/s41586-024-08253-8 (2025).

5 Hashemkhani, S., Vivekanand, V. S., Chopra, S. & Kubendran, R. Toward autonomous event-based sensorimotor control with supervised gait learning and obstacle avoidance for robot navigation. *Front Neurosci* **19**, 1492436 (2025).

6 Bonagiri, A., Biswas, D. & Chakravarthy, S. Coupled Memristor Oscillators for Neuromorphic Locomotion Control: Modeling and Analysis. *IEEE Trans Neural Netw Learn Syst* **35**, 8638-8652 (2024).

7 Yang, Y. *et al.* Firing feature-driven neural circuits with scalable memristive neurons for robotic obstacle avoidance. *Nat Commun* **15**, 4318 (2024).

8 Chen, L. *et al.* Spike timing-based coding in neuromimetic tactile system enables dynamic object classification. *Science* **384**, 660-665 (2024).

9 Dutta, S. *et al.* Programmable coupled oscillators for synchronized locomotion. *Nat Commun* **10**, 3299 (2019).
