# Supplementary material for: Artificial plateau neurons with in-situ spike-malleability for rhythmic quadrupedal locomotion
Source: Nat Commun. 2026 Apr 28;17:5801. doi: 10.1038/s41467-026-72428-2 (PMC13332192; doi:10.1038/s41467-026-72428-2)
Supplement: Supplementary file 3 — Description of Additional Supplementary Files [file 41467_2026_72428_MOESM3_ESM.pdf]

Description of Additional Supplementary Files

Supplementary Movie 1: Demonstration of the quadruped robot performing rhythmic air stepping in a real-world environment without ground contact.

Supplementary Movie 2: Simulation of the quadruped robot performing rhythmic air stepping in the Isaac Gym environment.

Supplementary Movie 3: Real-world demonstration of the quadruped robot walking on ground at a constant speed of approximately 0.15 m/s, showing stable trotting locomotion.

Supplementary Movie 4: Real-world demonstration of the quadruped robot adapting its walking speed on ground, transitioning from stationary stepping to cautious walking (0.15 m/s) and then to energetic walking (0.4 m/s), highlighting adaptive gait modulation.
