# Supplementary material for: Artificial plateau neurons with in-situ spike-malleability for rhythmic quadrupedal locomotion
Source: Nat Commun. 2026 Apr 28;17:5801. doi: 10.1038/s41467-026-72428-2 (PMC13332192; doi:10.1038/s41467-026-72428-2)
Supplement: Supplementary file 2 — Peer Review file [file 41467_2026_72428_MOESM2_ESM.pdf]

nature portfolio

Peer Review File

Artificial Plateau Neurons with In-situ Spike-Malleability for Rhythmic Quadrupedal Locomotion

Corresponding Author: Professor Bin Yu

This file contains all reviewer reports in order by version, followed by all author rebuttals in order by version.

Version 0:

Reviewer comments:

Reviewer #1

(Remarks to the Author)
Summary

The study “Artificial Plateau Neurons with In-situ Spike-Malleability for Rhythmic Quadrupedal Locomotion” presents a neuromorphic hardware framework inspired by biological central pattern generators (CPGs) to address the challenges of energy inefficiency and redundancy in robotic locomotion control. The authors design an artificial plateau neuron (PG-TS) that integrates a bistable plateau gate (PG) with a transient threshold switch (TS), thereby emulating the bistable depolarization and in-situ spike malleability characteristic of biological plateau neurons. This configuration enables the generation of amplitude- and phase-programmable rhythmic spike bursts without continuous external biasing.

The authors propose two neuromorphic circuit architectures: a dual-branch 2(PG-TS) circuit and a parallel 4PG-TS circuit. The 2(PG-TS) design achieves antagonistic extensor-flexor rhythmic spiking with exceptionally low power consumption (~141 pJ/spike), approaching the energy efficiency of biological neurons. The 4PG-TS configuration leverages multiply-accumulate (MAC)-like operations to broaden the modulation range of rhythmic spike encoding, thereby enhancing the circuit’s flexibility for more complex locomotion patterns.

Fabricated using wafer-scale MoS_2 and graphene, these circuits were validated experimentally both at the device level and in system-level applications. When implemented on a Unitree Go2 quadruped robot, distributed PG-TS oscillators generated Gaussian-filtered rhythmic spikes that directly drove proportional-derivative (PD) motor controllers. This eliminated the need for centralized computation and enabled stable, energy-efficient trotting gaits in both simulation (Isaac Gym) and physical experiments.

The authors highlight the potential of this neuromorphic hardware beyond robotics, with possible applications in prosthetics, motor rehabilitation, cardiac pacemakers, and in-memory computing for spiking neural networks (SNNs).

Decision: Recommend for publication with minor revisions

Detailed Review

This work demonstrates several notable merits:

Neuromorphic implementation: The PG-TS device introduces in-situ spike malleability combined with bistable depolarization, filling a critical gap in current artificial neuron designs. This enables direct emulation of CPG-like rhythmic dynamics, a capability previously inaccessible to conventional spiking neural networks or ANN-based approaches. However there are other works published on the spiking/bursting CPG network idea using bio-realistic neurons, such as Ref 1-4. Please add them to your references

Energy efficiency: The demonstrated energy dissipation of ~141 pJ/spike is highly competitive, approaching biological benchmarks and surpassing previously reported neuromorphic locomotion control circuits. This efficiency is particularly relevant for autonomous systems operating under strict energy constraints.

Wafer-scale fabrication: The successful integration of 2(PG-TS) and 4PG-TS circuits on a 2-inch wafer provides evidence of scalability and compatibility with semiconductor manufacturing processes. This is a critical step towards practical deployment of bio-inspired neuromorphic hardware.

Robotics application: The experimental validation on the Unitree Go2 quadruped robot represents a significant advance. The end-to-end, hardware-driven locomotion pipeline — from rhythmic spiking generation to motor actuation — illustrates the potential of compact neuromorphic circuits to replace conventional centralized computation in robotic control.

Nevertheless, the study also exhibits limitations to be addressed:

Immediate concerns The locomotion tasks are restricted primarily to trotting gaits. Demonstration across diverse gait patterns (walking, pacing, galloping) would provide stronger evidence of the generality and adaptability of the approach.

Long-term concerns:
The fabrication process, relying on CVD-grown MoS_2 and graphene, while effective at the research scale, may face challenges in large-scale industrial translation. While the circuits excel at rhythmic control, their applicability to broader neuromorphic tasks such as dynamic recognition, sensorimotor integration, or decision-making remains unexplored. Hybrid systems that integrate PG-TS circuits with higher-level computational modules could further enhance versatility.

In conclusion, this paper makes a significant contribution to the fields of neuromorphic engineering and bio-inspired robotics. By bridging device-level innovation with real-world robotic locomotion, the authors demonstrate a pathway toward energy-autonomous, hardware-centric intelligent systems. The artificial plateau neuron (PG-TS) offers a compact, efficient, and biologically faithful model of rhythmic neural dynamics, and its potential applications extend well beyond locomotion, including prosthetics, rehabilitation, and neuromorphic computation.

References
1. Hashemkhani, S., Vivekanand, V.S., Chopra, S. and Kubendran, R. (2025). Toward autonomous event-based sensorimotor control with supervised gait learning obstacle avoidance for robot navigation. Frontiers in Neuroscience, 19:1492436, doi: 10.3389/fnins.2025.1492436
2. Lele, A., Fang, Y., Ting, J., and Raychowdhury, A. (2021). An end-to-end spiking neural network platform for edge robotics: from event-cameras to central pattern generation. IEEE Trans. Cogn. Dev. Syst. 14, 1092–1103. doi: 10.1109/TCDS.2021.3097675
3. Aljalbout, E., Walter, F., Röhrbein, F., and Knoll, A. (2020). Task-independent spiking central pattern generator: a learning-based approach. Neural Process. Lett. 51, 2751–2764. doi: 10.1007/s11063-020-10224-9
4. Gutierrez-Galan, D., Dominguez-Morales, J. P., Perez-Peña, F., Jimenez-Fernandez, A., and Linares-Barranco, A. (2020). Neuropod: a real-time neuromorphic spiking CPG applied to robotics. Neurocomputing 381, 10–19. doi: 10.1016/j.neucom.2019.11.007

(Remarks on code availability)
The files in the URL have a broken link. The error message says file not found

Reviewer #2

(Remarks to the Author)
This paper presents the design, fabrication, and validation of a neuromorphic circuit that emulates the function of CPG neurons. The proposed artificial plateau neurons generate rhythmic spike bursts, mimicking the oscillatory and bursting dynamics observed in biological counterparts. The authors further demonstrate that rhythmic spike patterns can be harnessed to produce extensor–flexor activations with improved energy efficiency and reduced latency, enabling direct translation into movement of a quadrupedal robot. The work claims to address key challenges of redundant actuation and energy inefficiency in whole-body intelligent locomotion systems.

From a scientific perspective, this work is an innovative and technically sound approach toward realizing bio-inspired CPG functionality in integrated circuits. It is a meaningful step in advancing neuromorphic computing for autonomous robotic locomotion by achieving chip-level energy efficiency and scalable spike-based control. While the study shows functional verification, I have concerns about the scalability and robustness of the proposed design for deployment in real-world scenarios.

1. The claim of overall system-level energy efficiency is debatable. While the artificial neurons themselves exhibit ultra-low power consumption (as expected for an analog computing unit), the supporting circuitry in the current setup likely contributes significant overhead. In practice, DAC/ADC, biasing circuits, and other signal-conditioning components may be required to interface the neuron with control systems and actuators. The power consumption of these supporting components may exceed that of the neuron core by several orders of magnitude, potentially offsetting the reported energy gains. The authors should provide further justification and/or references discussing how the proposed neuron architecture could be integrated into a larger neuromorphic network, where the energy efficiency advantages may be more meaningfully realized at the system level.

2. The quadruped robot demonstration is not entirely convincing. In its current form, the robot does not perform actual locomotion but merely waves its legs in the air, limiting the experiment’s relevance to real-world control. While the artificial neuron mechanism is conceptually interesting, the control circuitry used here functions primarily as an oscillator that generates periodic signals with programmable frequency and amplitude. This can be readily reproduced through conventional analog or digital means. I understand that developing a fully closed-loop locomotion control system (as outlined in Supp. Fig. 15) may be beyond the scope of this work. However, the authors should consider redesigning the demonstration to more effectively validate the practical applicability, for example, by incorporating physical interaction with the environment or a simplified closed-loop test to substantiate its functional advantages.

3. It remains unclear how the proposed system would respond under varying real-world conditions such as walking, running, traversing slopes, operating on different surfaces, under varying loads. While the paper claims that the artificial neurons can adapt their behavior, this capability is not convincingly demonstrated. The current video demo appears equivalent to driving the robot with a pre-defined periodic pattern rather than an adaptive control scheme. It would also be helpful to clarify whether proprioceptive or sensory feedback is required to complete the control loop, and if so, how such feedback would be integrated to enable true behavioral adaptation.

4. The paper should provide a more comprehensive analysis of energy efficiency at the system level such as details regarding motor driving current, torque output, total energy consumption, and direct comparisons during actual locomotion. While the device demonstrates impressive energy efficiency at the chip level, the study does not yet establish that this translates into overall energy savings for the full quadrupedal locomotion system. Providing quantitative data or benchmarking against existing robotic control architectures would also strengthen the claim of system-level energy improvement.

5. What specific features of the proposed circuit necessitate a custom fabrication process? From a practical standpoint, could this design be implemented using standard mixed-signal processes, such as TSMC 130 nm. Additionally, the authors should elaborate on how they envision deploying this technology in practical robotic systems: as standalone neuromorphic chips or integrated modules within existing control IC.

6. Could you provide benchmarking of the reported activation latency of 5.62 μs against existing neuromorphic or conventional control circuits? This would help contextualize the performance advantage of the proposed architecture. Since the robot did not perform on-ground locomotion, it remains unclear whether the measured latency meaningfully impacts real-world control performance.

7. (Minor) In Table 1, the unit for biological neurons in the first column should be corrected from “pj” to “pJ.”

(Remarks on code availability)
-

Version 1:

Reviewer comments:

Reviewer #1

(Remarks to the Author)
The authors have thoroughly and comprehensively addressed all of the reviewer's comments and concerns. The revisions demonstrate a clear understanding of the feedback provided, with substantial improvements made to the methodology, experimental validation, and presentation of results. The additional analyses and clarifications have significantly strengthened the manuscript, resolving the previously identified issues. The paper now presents a well-structured, rigorous, and complete contribution to the field. Based on the quality of the revisions and the authors' detailed responses, I am satisfied that the manuscript meets the publication standards and recommend acceptance for publication.

(Remarks on code availability)
The github repo is well organized and the code is user friendly.

Reviewer #2

(Remarks to the Author)
The authors have satisfactorily addressed my concerns. I appreciate the inclusion of real-world experiments and the associated analysis, which gives me a better understanding of the proposed system. Overall, the manuscript is in good shape. This work presents an interesting proof of concept and is supported by comprehensive and well-executed analysis.

(Remarks on code availability)
-

Open Access This Peer Review File is licensed under a Creative Commons Attribution 4.0 International License, which permits use, sharing, adaptation, distribution and reproduction in any medium or format, as long as you give appropriate credit to the original author(s) and the source, provide a link to the Creative Commons license, and indicate if changes were made.
In cases where reviewers are anonymous, credit should be given to 'Anonymous Referee' and the source.
The images or other third party material in this Peer Review File are included in the article's Creative Commons license, unless indicated otherwise in a credit line to the material. If material is not included in the article's Creative Commons license and your intended use is not permitted by statutory regulation or exceeds the permitted use, you will need to obtain permission directly from the copyright holder.
To view a copy of this license, visit https://creativecommons.org/licenses/by/4.0/

Point-by-Point Response
to All Reviewers' Comments

Dear Editor and Reviewers,

We sincerely appreciate your spending valuable time reviewing our manuscript and put together the feedback to help us significantly improve our manuscript. We are particularly encouraged by Reviewer 1's positive assessment of our work's novelty, energy efficiency, and successful robotic demonstration. **We consider it necessary to provide a detailed, point-by-point response to all the reviewers' comments and to clarify some critical issues.** We submit this Response along with the revised manuscript and Supporting Information with colored marks indicating all the changes.

Once again, thank you very much for reviewing!

--

Response to Referee #1's Review

--

The study "Artificial Plateau Neurons with In-situ Spike-Malleability for Rhythmic Quadrupedal Locomotion" presents a neuromorphic hardware framework inspired by biological central pattern generators (CPGs) to address the challenges of energy inefficiency and redundancy in robotic locomotion control. The authors design an artificial plateau neuron (PG-TS) that integrates a bistable plateau gate (PG) with a transient threshold switch (TS), thereby emulating the bistable depolarization and in-situ spike malleability characteristic of biological plateau neurons. This configuration enables the generation of amplitude- and phase-programmable rhythmic spike bursts without continuous external biasing.

The authors propose two neuromorphic circuit architectures: a dual-branch 2(PG-TS) circuit and a parallel 4PG-TS circuit. The 2(PG-TS) design achieves antagonistic extensor-flexor rhythmic spiking with exceptionally low power consumption (~141

pJ/spike), approaching the energy efficiency of biological neurons. The 4PG-TS configuration leverages multiply-accumulate (MAC)-like operations to broaden the modulation range of rhythmic spike encoding, thereby enhancing the circuit's flexibility for more complex locomotion patterns.

Fabricated using wafer-scale MoS_2 and graphene, these circuits were validated experimentally both at the device level and in system-level applications. When implemented on a Unitree Go2 quadruped robot, distributed PG-TS oscillators generated Gaussian-filtered rhythmic spikes that directly drove proportional-derivative (PD) motor controllers. This eliminated the need for centralized computation and enabled stable, energy-efficient trotting gaits in both simulation (Isaac Gym) and physical experiments.

The authors highlight the potential of this neuromorphic hardware beyond robotics, with possible applications in prosthetics, motor rehabilitation, cardiac pacemakers, and in-memory computing for spiking neural networks (SNNs).

Our response:

We thank Reviewer #1 for the positive and constructive evaluation of our manuscript. We appreciate the reviewer's recognition of the novelty of the artificial plateau neuron concept, the demonstrated energy efficiency approaching biological benchmarks, the wafer-scale fabrication, and the successful end-to-end robotic implementation.

We carefully address all the reviewer's comments. We add the suggested references on bio-realistic spiking and bursting CPG networks to better position our work within the existing literature, clarified the distinctions between prior software-based or network-level CPG implementations and our device-level neuromorphic realization, and correct the code availability issue by updating the repository link. All revisions are highlighted in the revised manuscript and Supporting Information.

*Comment #1: *"However there are other works published on the spiking/bursting CPG network idea using bio-realistic neurons, such as Ref 1-4. Please add them to your*

*references."**

Our Response:

We sincerely thank the reviewer for this valuable suggestion. We agree that situating our work within the broader context of spiking/bursting CPGs is important. Accordingly, we add the four recommended references (Hashemkhani et al., 2025; Lele et al., 2021; Aljalbout et al., 2020; Gutierrez-Galan et al., 2020) to the Introduction sections of the revised manuscript. While these works focus on network-level or software-based spiking CPG implementations using bio-realistic neuron models, our work differs fundamentally by realizing CPG-like rhythmic dynamics at the device and circuit level, enabling ultra-low-power, bias-free operation and direct hardware-to-actuator interfacing.

Text Revision:

Based on the reviewer's helpful suggestion to the references, we revise the manuscript accordingly on page 12:

We add references to the manuscript on page 12, lines 426-433, lines 445-447:

5 Lele, A., Fang, Y., Ting, J. & Raychowdhury, A. An End-to-End Spiking Neural Network Platform for Edge Robotics: From Event-Cameras to Central Pattern Generation. *IEEE Trans. Cognit. Dev. Syst.* **14**, 1092-1103 (2022).

6 Gutierrez-Galan, D., Dominguez-Morales, J. P., Perez-Peña, F., Jimenez-Fernandez, A. & Linares-Barranco, A. Neuropod: A real-time neuromorphic spiking CPG applied to robotics. *Neurocomputing* **381**, 10-19 (2020).

7 Aljalbout, E., Walter, F., Röhrbein, F. & Knoll, A. Task-Independent Spiking Central Pattern Generator: A Learning-Based Approach. *Neural Processing Letters* **51**, 2751-2764 (2020).

13 Hashemkhani, S., Vivekanand, V. S., Chopra, S. & Kubendran, R. Toward autonomous event-based sensorimotor control with supervised gait learning and

obstacle avoidance for robot navigation. *Front Neurosci* **19**, 1492436 (2025).

Comment #2: "Immediate concerns: The locomotion tasks are restricted primarily to trotting gaits. Demonstration across diverse gait patterns (walking, pacing, galloping) would provide stronger evidence of the generality and adaptability of the approach."

Our Response:

We thank the reviewer for the constructive suggestion to strengthen the evidence for our approach's generality and adaptability by demonstrating diverse gait patterns. In direct response, we expand our experimental validation beyond the initial focus. The revised manuscript now presents results from successful on-ground locomotion experiments with the Unitree Go2 quadruped robot, which demonstrate both stable pattern generation and gait adaptability.

Stable Gait Generation and Multi-Gait Programmability: We first validate the system's capability for steady, coordinated locomotion. As shown in the new Fig. R1.1 a-d, the robot achieves stable forward trotting at 0.15 m/s using a diagonal synchronization strategy driven by our PG-TS oscillators. The integrated data chain—from the hardware-generated rhythmic spike bursts (Fig. R1.1 b), to the resulting joint angle trajectories (Fig. R1.1 c), and the spatiotemporal event heatmap (Fig. R1.1 d) confirms the translation from neuromorphic rhythm generation to effective physical locomotion (Supplementary Video 3). Furthermore, to explicitly showcase programmability for different patterns, Fig. R1.2 demonstrates that by modulating the PG-TS circuit parameters, we can generate distinct rhythmic outputs corresponding to walking and pacing gaits.

In-Situ Gait Adaptation: Critically, we demonstrate that the generated gait is not a fixed pattern but can be dynamically modulated. By leveraging the *in-situ* spike malleability of the PG-TS neurons through external inputs (e.g., optical bias), we command the robot to execute an adaptive walking sequence (Fig. R1.3). As shown in Fig. R1.1 e-h, the robot smoothly transitions its behavior from stationary stepping to cautious walking (0.15 m/s) and then to more energetic walking (0.4 m/s). The

corresponding evolution of the spike patterns (Fig. R1.1 f), joint angles (Fig. R1.1 g), and their spatiotemporal coordination (Fig. R1.1 h) illustrate continuous behavioral modulation (Supplementary Video 4).

These new results collectively provide stronger evidence for the generality and adaptability of our approach. They show that the PG-TS hardware can be programmed for different gaits and can continuously adapt its output, underscoring its potential for versatile locomotor control.

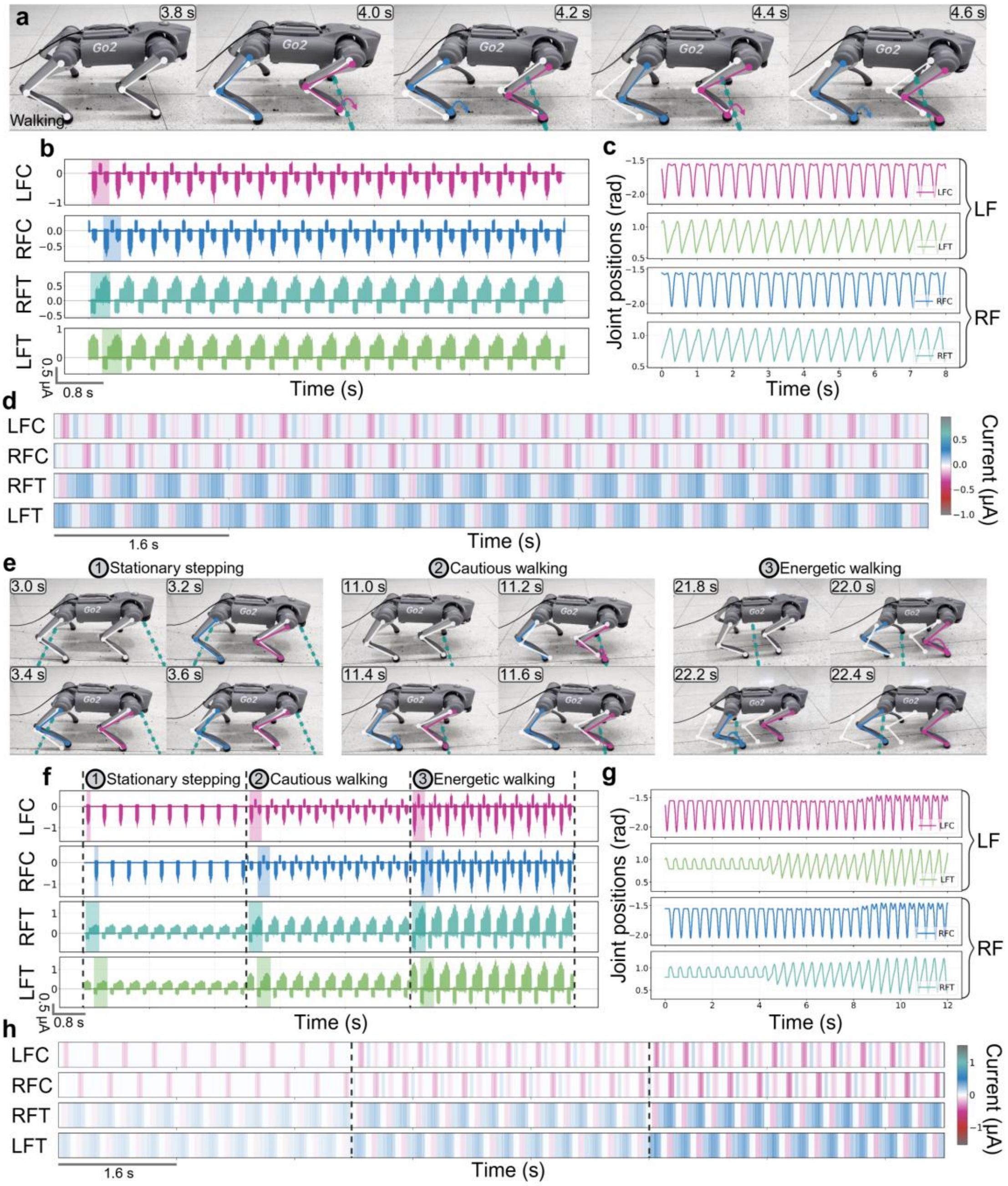

Fig. R1.1 On-ground adaptive locomotion of the quadruped robot. a, Physical demonstration of the Unitree Go2 robot performing steady on-ground trotting. White limb guides indicate the initial stance. Pink and blue guides highlight the motion of the RF and RH limbs, respectively, under a diagonal synchronization strategy. b, Rhythmic spike bursts from the four joint actuator groups. c, Actual joint angle oscillation signals. d, Heatmap of the rhythmic spike events, illustrating the spatiotemporal pattern for coordinated locomotion. e, Physical demonstration of adaptive on-ground walking. The adaptive gait sequence consists of three phases: 1. Stationary stepping, 2. Cautious walking, 3. Energetic walking. f-h, Adaptive rhythmic spike bursts modulating across the three phases (f), The actual joint angle oscillation curves (g), Heatmap of the spike events across the three phases, showing the transition in the spatiotemporal pattern.

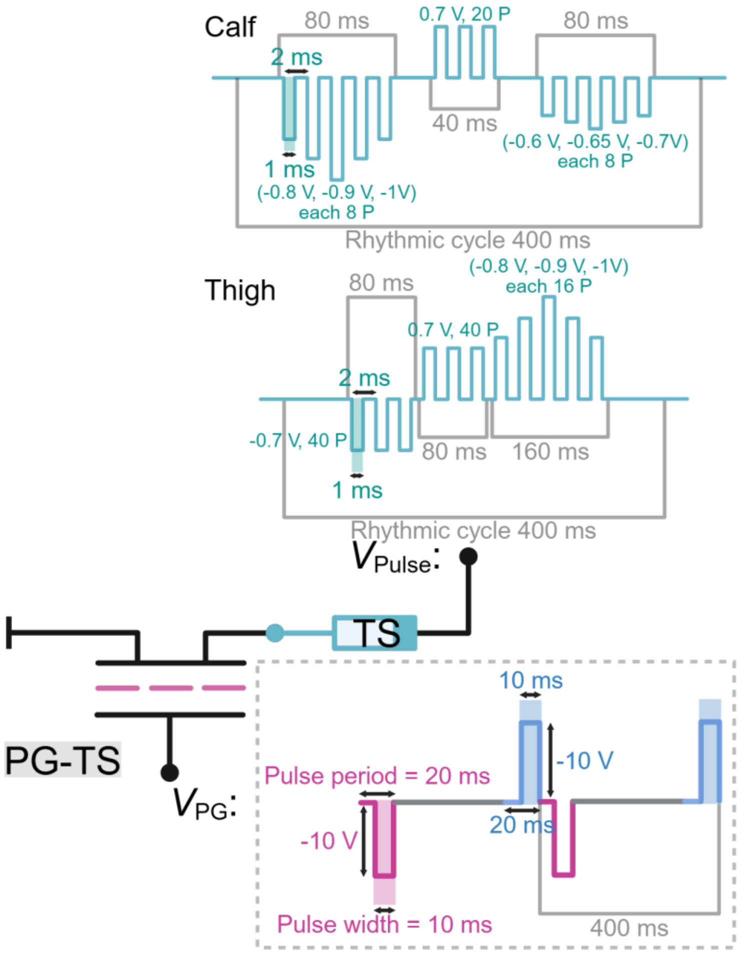

Fig. R1.2 Control voltages waveform for rhythmic spike output. The rhythmic period is 400 ms. Within each period, V_{PG} is a periodic depolarizing/hyperpolarizing voltage with 50% duty cycle and an amplitude of 10 V. V_{Pulse} (50% duty cycle and a period of 2 ms) for the calf joints, V_{Pulse} exhibits a staircase voltage that rises and then falls between 60–140 ms and 260–340 ms, and a constant 0.7 V input between 180–220 ms. For the thigh joints, V_{Pulse} provides a constant –0.7 V input between 40–120 ms, a constant 0.7 V input between 120–200 ms, and a staircase voltage that rises and then falls between 200–400 ms.

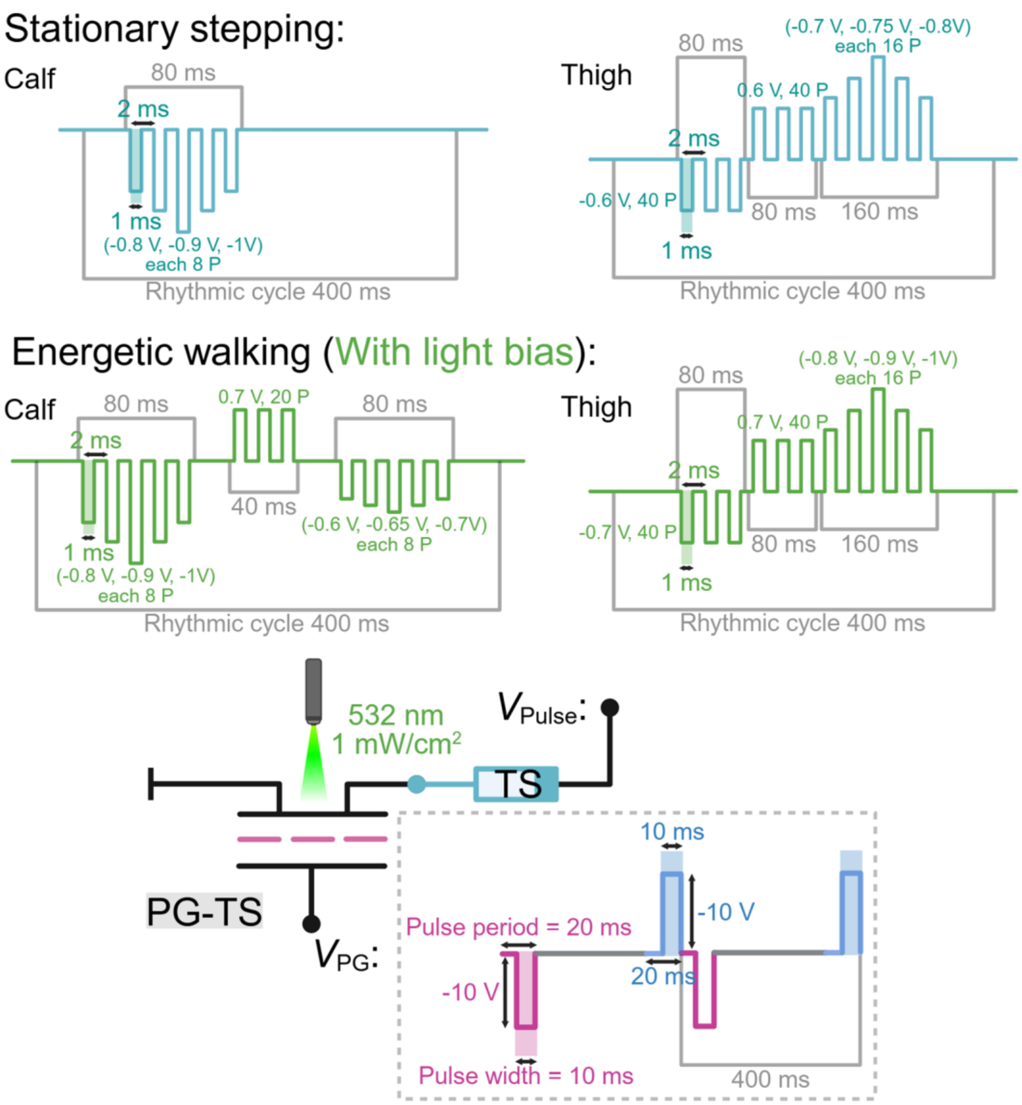

Fig. R1.3 Control voltages waveform for the three-phase locomotion. V_{PG} is a periodic depolarizing/hyperpolarizing voltage with 50% duty cycle and an amplitude of 10 V. Compared to steady slow walking, the stationary-stepping phase lacks the reverse-oscillation input stage and the small-amplitude staircase input stage for calf. Leveraging 2D optical response of the plateau neurons, a visible-light (532 nm) bias of 1 mW/cm^2 applied under the pulse conditions for slow walking. The rhythmic spikes output exhibit photo-adaptive enhancement, thereby meeting the large-amplitude swing required for the robot's energetic walking.

Text Revision:

Based on the reviewer's helpful suggestion to the quadruped walks, we revise the manuscript accordingly on page 8, page 21:

We add the supplementary experimental results figure and corresponding caption in Fig5 on page 20, lines 620–631:

(**Fig 5** here refers to **Fig. R1.1** mentioned above and would not be listed again.)

We add related discussion to the manuscript on page 8, lines 275-285; lines 291-303:

On-ground adaptive locomotion of the quadruped robot

We first validate the system's capability for steady, coordinated trotting. As shown in Fig. 5a, the robot achieved stable forward locomotion at a speed of 0.15 m/s using the diagonal synchronization strategy (Supplementary Video 3). The underlying control signals are presented in Fig. 5b–d: the rhythmic spike bursts generated by the plateau neurons for the LFC, RFC, RFT, and LFT joints (Fig. 5b); the corresponding control voltage are shown in Supplementary Fig. 17, where the left and right limb joints exhibit anti-phase spike output with a 200-ms phase offset. The Gaussian-filtered joint angle trajectories that drove the PD controllers (Fig. 5c), together with a heatmap visualizing the precise spatiotemporal pattern of the spike events that orchestrate the gait (Fig. 5d), complete an integrated data chain that confirms translation from

plateau-neuron rhythm generation to effective physical locomotion.

Beyond steady-state operation, we implement adaptive walking to demonstrate the tunable capability of the plateau neuron hardware. By modulating input voltage to the plateau neuron circuits and enhancing the rhythmic spiking under optical bias (Supplementary Fig. 20), we command the robot to execute a three-phase adaptive sequence: stationary stepping, cautious walking (0.15 m/s), and energetic walking (0.4 m/s) (Fig. 5e) (Supplementary Video 4). The resulting adaptive rhythmic spike bursts, which maintain the 200-ms phase difference between left and right limbs, are shown in Fig. 5f. The smoothly transitioning joint-angle profiles (Fig. 5g) and the evolving spatiotemporal heatmap of spike bursts (Fig. 5h) illustrate the system's capacity for continuous behavioral modulation based on external inputs. This demonstrates that the *in-situ* spike malleability of plateau neurons harnessed for dynamic gait adaptation. Taken together, these experiments provide evidence that our neuromorphic CPG hardware can generate control signals sufficient for effective on-ground locomotion and exhibits the inherent programmability required for adaptive behavior.

We present the expanded Abstract in the main text on page 1, lines 30–32:

Critically, the system achieves stable on-ground locomotion and demonstrates adaptive gait transitions in real-world environments.

We revise the wording in the Introduction on page 3, lines 87–90:

We validate stable trotting through simulations (Isaac Gym) and physical experiments, including on-ground walking and adaptive gait modulation via external inputs, with programmable spiking dynamics enabling rhythmic locomotion.

We add text to the Discussion on page 9, lines 324–327:

The system successfully executes stable on-ground locomotion and exhibits adaptive walking through direct modulation of plateau neuron's operating point, confirming its potential for real-world deployment in dynamic environments.

The supporting information is revised accordingly on page 20, page 23:

We add Supplementary Figure 17 titled: "Control voltages waveform for rhythmic spike output." on page 20, lines 190-197:

(**Supplementary Figure 17** here refers to **Fig. R1.2** mentioned above and would not be listed again.)

We add Supplementary Figure 17 titled: "Control voltages waveform for the three-phase locomotion." on page 23, lines 221-228:

(**Supplementary Figure 20** here refers to **Fig. R1.3** mentioned above and would not be listed again.)

Comment #3: "The fabrication process, relying on CVD-grown MoS_2 and graphene, while effective at the research scale, may face challenges in large-scale industrial translation. While the circuits excel at rhythmic control, their applicability to broader neuromorphic tasks such as dynamic recognition, sensorimotor integration, or decision-making remains unexplored. Hybrid systems that integrate PG-TS circuits with higher-level computational modules could further enhance versatility."

Our Response:

We thank the reviewer for these insightful comments regarding long-term scalability and the potential for broader applications. We appreciate the opportunity to discuss these forward-looking perspectives.

On the scalability of the CVD-based fabrication process: We fully agree that the path to large-scale industrial integration for any emerging material system requires dedicated development. Our current work demonstrates the functional potential and foundational energy efficiency of this hardware primitive at the research scale. We concur with the reviewer that its future translation would likely follow a heterogeneous

path, where the PG-TS core (optimized for its unique function) is combined with mainstream silicon CMOS. We incorporate a brief discussion on this integration pathway in the revised manuscript to acknowledge this point.

On applicability to broader neuromorphic tasks: This is an excellent point that highlights a promising direction for future research. The core computational properties of the PG-TS device—*in-situ* spike malleability and bistable dynamics—are indeed relevant for temporal signal processing beyond rhythmicity. While the present study is focused on establishing its efficacy for locomotion control, we now explicitly mention in the Discussion that these device characteristics "For practical deployment, a viable path lies in heterogeneous integration, where such neuromorphic cores can be combined with digital silicon CMOS. Furthermore, the artificial plateau neurons with in-situ spike-malleability hold promise for applications in low-power cardiac pacemaker design and emerging SNN in-memory computing architectures." We frame this as an exciting future direction motivated by the reviewer's suggestion.

Text Revision:

Based on the reviewer's helpful suggestion, we revise the manuscript accordingly on page 9:

We add code availability to the manuscript on page 9, line 330-332:

For practical deployment, a viable path lies in heterogeneous integration, where such neuromorphic cores can be combined with digital silicon CMOS.

Comment #4: "The files in the URL have a broken link. The error message says file not found."

Our Response:

We sincerely apologize for this oversight. The link to the code checked and corrected. It is now fully accessible.

Text Revision:

Based on the reviewer's helpful suggestion to the code availability, we revise the manuscript accordingly on page 12:

We add code availability to the manuscript on page 12, line 415-416:

The code supporting the configuration of the robot in this study is available at https://github.com/chaiqingao/go2_control with unrestricted access.

Response to Referee #2's Review

**"From a scientific perspective, this work is an innovative and technically sound approach toward realizing bio-inspired CPG functionality in integrated circuits. It is a meaningful step in advancing neuromorphic computing for autonomous robotic locomotion by achieving chip-level energy efficiency and scalable spike-based control. While the study shows functional verification, I have concerns about the scalability and robustness of the proposed design for deployment in real-world scenarios."*

Our Response:

We thank Reviewer #2 for the thoughtful and detailed review, and for recognizing the technology and innovation of our neuromorphic CPG hardware. We appreciate the reviewer's insightful comments regarding system-level energy efficiency, robustness, scalability, and practical deployment in real-world robotic scenarios.

In response, we substantially clarify the scope and intent of this work and expanded the discussion on system-level considerations. Specifically, we add quantitative analysis and discussion on motor driving energy, control latency, and the role of supporting circuitry, clarified the distinction between open-loop gait generation and closed-loop adaptive control, and discussed how sensory feedback and higher-level control modules can be integrated with the proposed PG-TS circuits. We expand the

comparison with conventional and neuromorphic control approaches to better contextualize the reported latency and energy advantages. These revisions strengthen the practical relevance of the proposed architecture while remaining consistent with the focus of this study on device-level neuromorphic innovation.

Comment #1: "The claim of overall system-level energy efficiency is debatable. While the artificial neurons themselves exhibit ultra-low power consumption (as expected for an analog computing unit), the supporting circuitry in the current setup likely contributes significant overhead. In practice, DAC/ADC, biasing circuits, and other signal-conditioning components may be required to interface the neuron with control systems and actuators. The power consumption of these supporting components may exceed that of the neuron core by several orders of magnitude, potentially offsetting the reported energy gains. The authors should provide further justification and/or references discussing how the proposed neuron architecture could be integrated into a larger neuromorphic network, where the energy efficiency advantages may be more meaningfully realized at the system level."

Our Response:

We thank the reviewer for this insightful comment, which rightly highlights the critical distinction between device-level efficiency and meaningful system-level advantage. We fully agree that the power consumption of peripheral interface circuitry (DAC/ADC, biasing, etc.) is a paramount consideration. To address this, we elaborate on a concrete system integration framework founded on the established neuromorphic engineering principle of heterogeneous specialization.

Our envisioned solution, illustrated in Fig. R2.1, employs a co-designed architecture where the ultra-low-power PG-TS core array functions as a dedicated, analog-mixed-signal "Rhythm Engine" for generating coordinated motor patterns. A separate digital processor handles higher-level, non-periodic tasks (e.g., sensor fusion and adaptation logic). This partition ensures each subsystem operates at its peak

efficiency for its specific function. The system-level efficiency of this architecture emerges through scaling. In a full-scale implementation, the fixed overhead of essential shared support circuits is amortized across hundreds or thousands of PG-TS cores[1]. Consequently, the per-core peripheral power becomes negligible, and the total system energy for rhythm generation is ultimately dominated by the intrinsic efficiency of the computational primitives (141.37 pJ/spike). This scaling principle, where core efficiency dictates system efficiency in large arrays, is well-supported in the literature[2,3].

Technologically, this vision is grounded in feasible pathways. The wafer-scale compatibility of 2D material heterostructures for the PG-TS core, combined with emerging 2.5D/3D integration schemes for combining analog neuromorphic and digital logic tiers, provides a clear route to realization[4,5]. Thus, while our present study validates the core computational unit, the revised manuscript now includes a justified roadmap for translating its device-level performance into practical system-level gains.

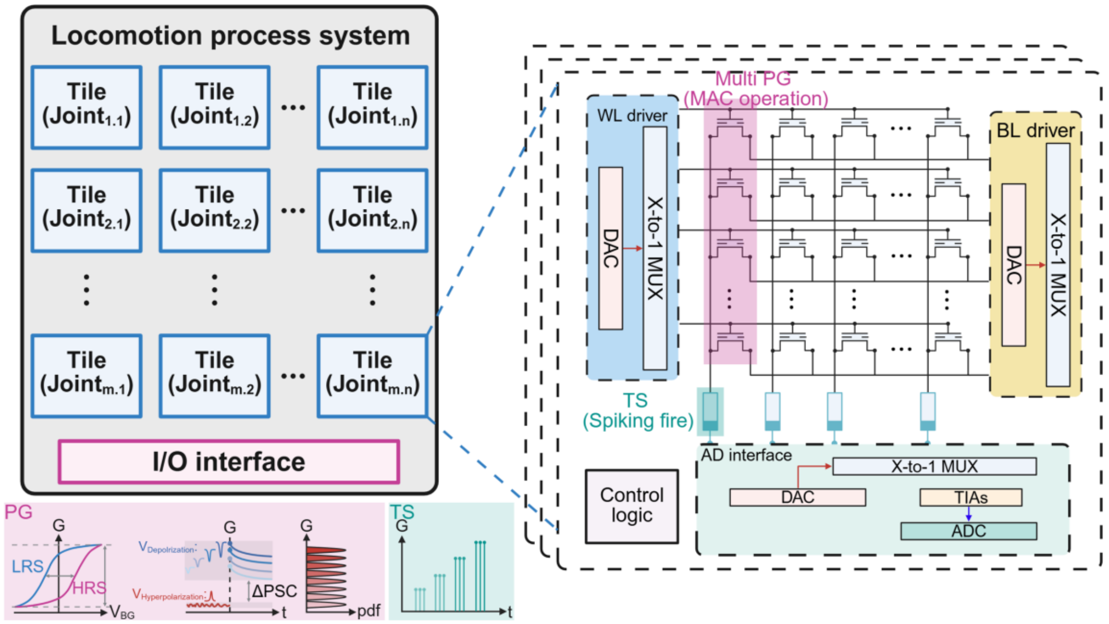

Fig. R2.1 Conceptual architecture for a scalable neuromorphic locomotion control system. The overall locomotion control system comprises multiple independent Tiles, each responsible for controlling a specific set of robotic joints. Each Tile integrates the PG array with parallel TS output channels. It also incorporates common peripheral circuits essential for system function, including inverting control circuits, TIA, analog-

to-digital/digital-to-analog converters (ADC/DAC), and multiplexers (MUX). c, Building upon the experimentally validated 4PG-TS circuit (lower left), the PG array can be scaled to a larger configuration. In such an array, the output rhythmic spiking intensity can be precisely modulated through MAC operations and linear regression, enabled by programming the weight of each PG via its V_{PG} voltage. This scalable, multi-channel architecture is particularly suited for controlling complex joint assemblies (e.g., finger joints), where a single macro-joint is driven by multiple micro-joints. The parallel outputs of a (Multi PG)-TS Tile can generate synchronized rhythmic spikes to coordinate the motion of all sub-joints simultaneously.

Text Revision:

Based on the reviewer's helpful suggestion to the system-level efficiency, we revise the manuscript accordingly on page 9:

We add related discussion to the manuscript on page 9, lines 318-322:

Crucially, the pathway to system-level efficiency lies in scalable integration. As conceptualized in Supplementary Figure. 21, these PG-TS cores can be organized into parallel arrays, where the fixed overhead of peripheral circuits is amortized across many cores, ensuring that the intrinsic low energy per spike (~141 pJ/spike) dominates the system's energy budget for rhythm generation.

The supporting information is revised accordingly on page 24:

We add Supplementary Figure 17 titled: "Conceptual architecture for a scalable neuromorphic locomotion system." on page 24, lines 230-242:

(**Supplementary Figure 21** here refers to **Fig. R2.1** mentioned above and would not be listed again.)

Comment #2: "The quadruped robot demonstration is not entirely convincing. In its current form, the robot does not perform actual locomotion but merely waves its legs in the air, limiting the experiment's relevance to real-world control. While the artificial neuron mechanism is conceptually interesting, the control circuitry used here functions primarily as an oscillator that generates periodic signals with programmable frequency and amplitude. This can be readily reproduced through conventional analog or digital means. I understand that developing a fully closed-loop locomotion control system (as outlined in Supp. Fig. 15) may be beyond the scope of this work. However, the authors should consider redesigning the demonstration to more effectively validate the practical applicability, for example, by incorporating physical interaction with the environment or a simplified closed-loop test to substantiate its functional advantages."

Our Response:

We thank the reviewer for this insightful comment, which rightly emphasizes the need to demonstrate practical locomotion control. In direct response, we redesign our experiment to validate on-ground locomotion.

We successfully implement and characterize stable on-ground locomotion with the Unitree Go2 quadruped robot. As shown in the Fig. R2.2 a-d, the system achieves steady forward trotting at 0.15 m/s using our distributed plateau neurons as the CPGs. The control voltages are shown in Fig. R2.3, where the left and right limb joints exhibit anti-phase spike output with a 200 ms phase offset. The integrated data chain—from the hardware-generated rhythmic spike bursts for all actuated joints (Fig. R2.2 b), to the corresponding Gaussian-filtered joint angle positions that drive PD controllers (Fig. R2.2 c), and the spatiotemporal heatmap of the rhythmic spike events (Fig. R2.2 d)—confirms the translation of plateau neuron dynamics into effective physical locomotion. This result is comprehensively documented in the updated Supplementary Video 3.

This new experiment moves beyond an oscillator demonstration, proving that our system can generate the precise control signals required for stable, coordinated limb movement in a real-world environment. We emphasize that the proposed PG-TS neuron

is not intended to replace high-level planning or adaptive control modules, but to serve as a hardware-efficient rhythmic primitive analogous to biological spinal CPGs. The system's capability for adaptive modulation in response to external inputs is separately demonstrated and addressed in our response to Comment 3 below.

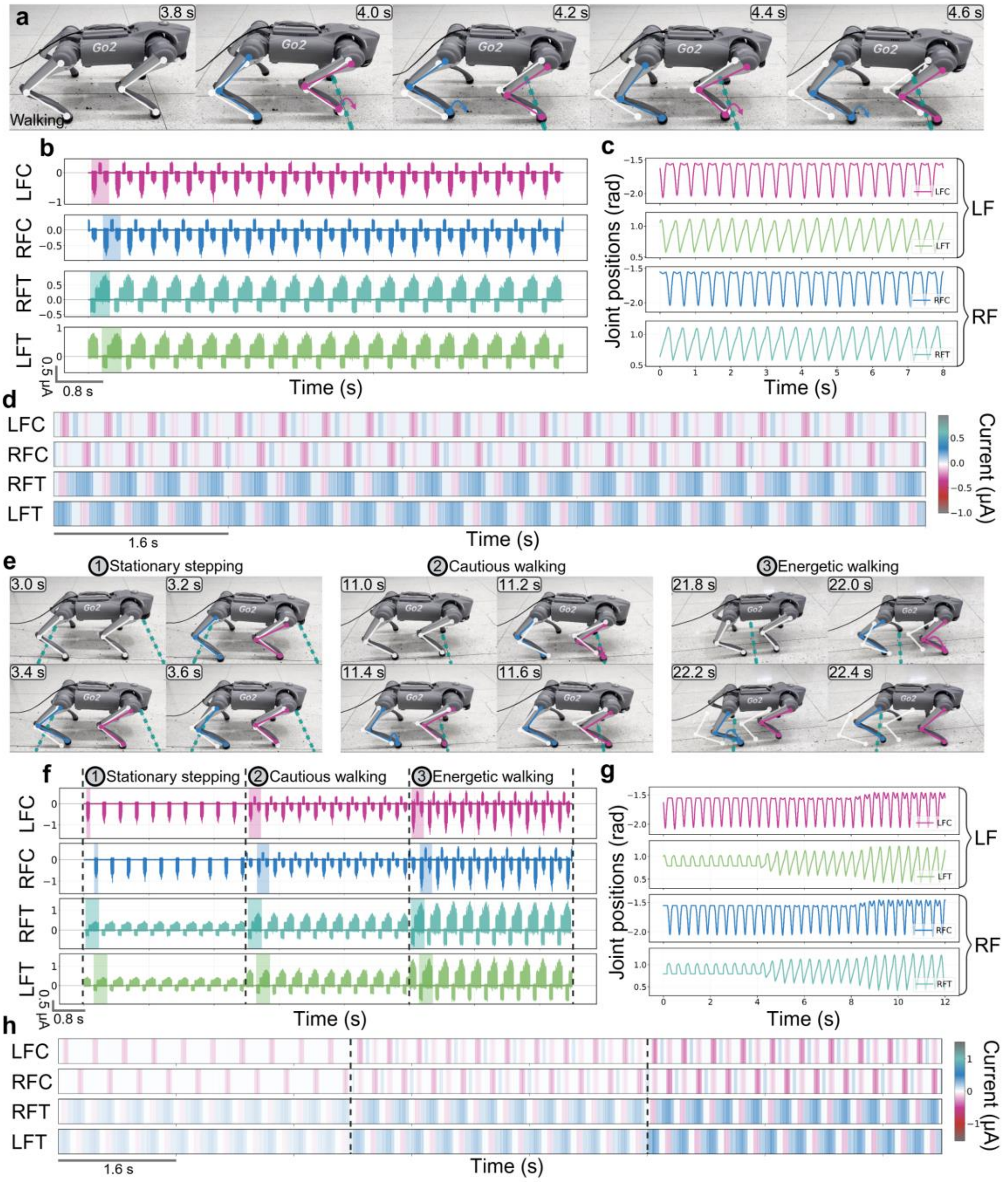

Fig. R2.2 On-ground adaptive locomotion of the quadruped robot. a, Physical demonstration of the Unitree Go2 robot performing steady on-ground trotting. White limb guides indicate the initial stance. Pink and blue guides highlight the motion of the

RF and RH limbs, respectively, under a diagonal synchronization strategy. b, Rhythmic spike bursts from the four joint actuator groups. c, Actual joint angle oscillation signals. d, Heatmap of the rhythmic spike events, illustrating the spatiotemporal pattern for coordinated locomotion. e, Physical demonstration of adaptive on-ground walking. The adaptive gait sequence consists of three phases: 1. Stationary stepping, 2. Cautious walking, 3. Energetic walking. f-h, Adaptive rhythmic spike bursts modulating across the three phases (f), The actual joint angle oscillation curves (g), Heatmap of the spike events across the three phases, showing the transition in the spatiotemporal pattern.

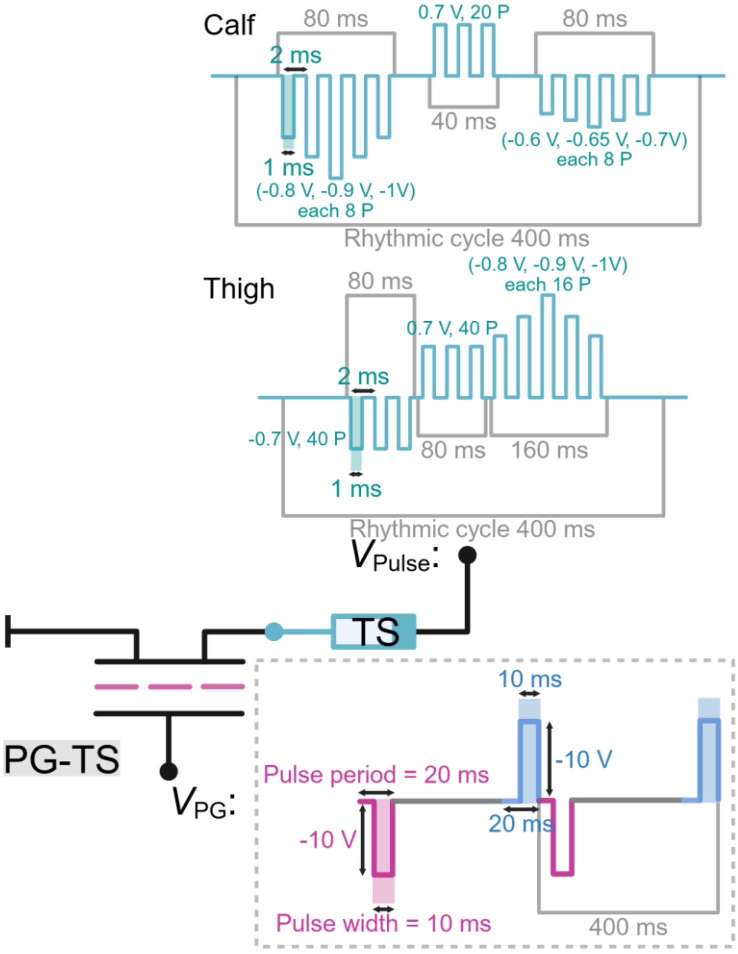

Fig. R2.3 Control voltages waveform for rhythmic spike output. The rhythmic period is 400 ms. Within each period, V_{PG} is a periodic depolarizing/hyperpolarizing voltage with 50% duty cycle and an amplitude of 10 V. V_{Pulse} (50% duty cycle and a period of 2 ms) for the calf joints, V_{Pulse} exhibits a staircase voltage that rises and then

falls between 60–140 ms and 260–340 ms, and a constant 0.7 V input between 180–220 ms. For the thigh joints, V_{Pulse} provides a constant –0.7 V input between 40–120 ms, a constant 0.7 V input between 120–200 ms, and a staircase voltage that rises and then falls between 200–400 ms.

Text Revision:

Based on the reviewer's helpful suggestion to the quadruped walks, we revise the manuscript accordingly on page 8, page 21:

We add related discussion to the manuscript on page 8, lines 275-285:

On-ground adaptive locomotion of the quadruped robot

We first validate the system's capability for steady, coordinated trotting. As shown in Fig. 5a, the robot achieved stable forward locomotion at a speed of 0.15 m/s using the diagonal synchronization strategy (Supplementary Video 3). The underlying control signals are presented in Fig. 5b–d: the rhythmic spike bursts generated by the plateau neurons for the LFC, RFC, RFT, and LFT joints (Fig. 5b); the corresponding control voltage are shown in Supplementary Fig. 17, where the left and right limb joints exhibit anti-phase spike output with a 200-ms phase offset. The Gaussian-filtered joint angle trajectories that drove the PD controllers (Fig. 5c), together with a heatmap visualizing the precise spatiotemporal pattern of the spike events that orchestrate the gait (Fig. 5d), complete an integrated data chain that confirms translation from plateau-neuron rhythm generation to effective physical locomotion.

We add the supplementary experimental results figure and corresponding caption in Fig5 on page 20, lines 620–631:

(**Fig 5** here refers to **Fig. R2.2** mentioned above and would not be listed again.)

The supporting information is revised accordingly on page 20.

We add Supplementary Figure 17 titled: "Control voltages waveform for rhythmic spike output." on page 20, lines 187-194.

(**Supplementary Figure 17** here refers to **Fig. R2.3** mentioned above and would not be listed again.)

Comment #3: "It remains unclear how the proposed system would respond under varying real-world conditions such as walking, running, traversing slopes, operating on different surfaces, under varying loads. While the paper claims that the artificial neurons can adapt their behavior, this capability is not convincingly demonstrated. The current video demo appears equivalent to driving the robot with a pre-defined periodic pattern rather than an adaptive control scheme. It would also be helpful to clarify whether proprioceptive or sensory feedback is required to complete the control loop, and if so, how such feedback would be integrated to enable true behavioral adaptation."

Our Response:

We thank the reviewer for this important comment regarding the system's adaptability to dynamic real-world conditions. In response, we conduct new experiments that explicitly demonstrate adaptive locomotion control through direct modulation of our neuromorphic CPGs.

As shown in Fig. R2.2 e-h and Supplementary Video 4, we command the Unitree Go2 robot to perform a three-phase adaptive walking sequence—transitioning from stationary stepping to cautious (0.15 m/s) and then to energetic walking (0.4 m/s)—by applying real-time modulations to the plateau neurons' control parameters (V_{Pulse} and an optical bias of 532 nm, 1 mW/cm^2). The resulting evolution in spike burst patterns (Fig. R2.2 f), joint angle trajectories (Fig. R2.2 g), and spatiotemporal coordination (Fig. R2.2 h) confirms that the motor output is not a fixed pattern but can be continuously shaped by external inputs.

This adaptability stems from the intrinsic in-situ spike malleability of the PG-TS neuron. While the current study demonstrates open-loop modulation, it establishes a

direct pathway for closed-loop sensorimotor integration. Future systems can encode proprioceptive or exteroceptive feedback (e.g., from joint encoders, IMUs, or pressure sensors) as analogous low-dimensional modulation signals to enable true behavioral adaptation. We expand the discussion to elaborate on this vision for integrating sensory feedback within this neuromorphic control framework.

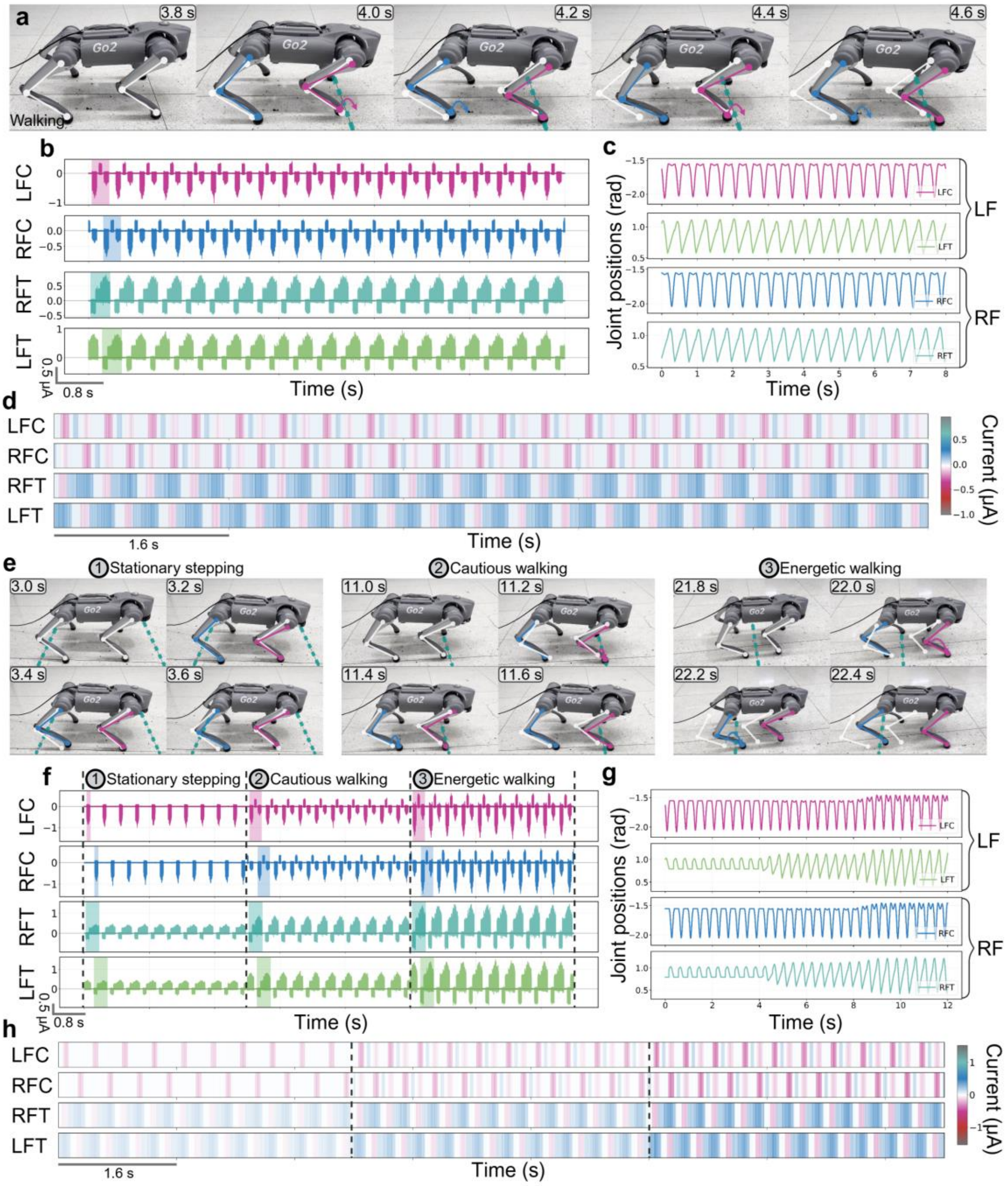

Fig. R2.2 On-ground adaptive locomotion of the quadruped robot. a, Physical demonstration of the Unitree Go2 robot performing steady on-ground trotting. White

limb guides indicate the initial stance. Pink and blue guides highlight the motion of the RF and RH limbs, respectively, under a diagonal synchronization strategy. b, Rhythmic spike bursts from the four joint actuator groups. c, Actual joint angle oscillation signals. d, Heatmap of the rhythmic spike events, illustrating the spatiotemporal pattern for coordinated locomotion. e, Physical demonstration of adaptive on-ground walking. The adaptive gait sequence consists of three phases: 1. Stationary stepping, 2. Cautious walking, 3. Energetic walking. f-h, Adaptive rhythmic spike bursts modulating across the three phases (f), The actual joint angle oscillation curves (g), Heatmap of the spike events across the three phases, showing the transition in the spatiotemporal pattern.

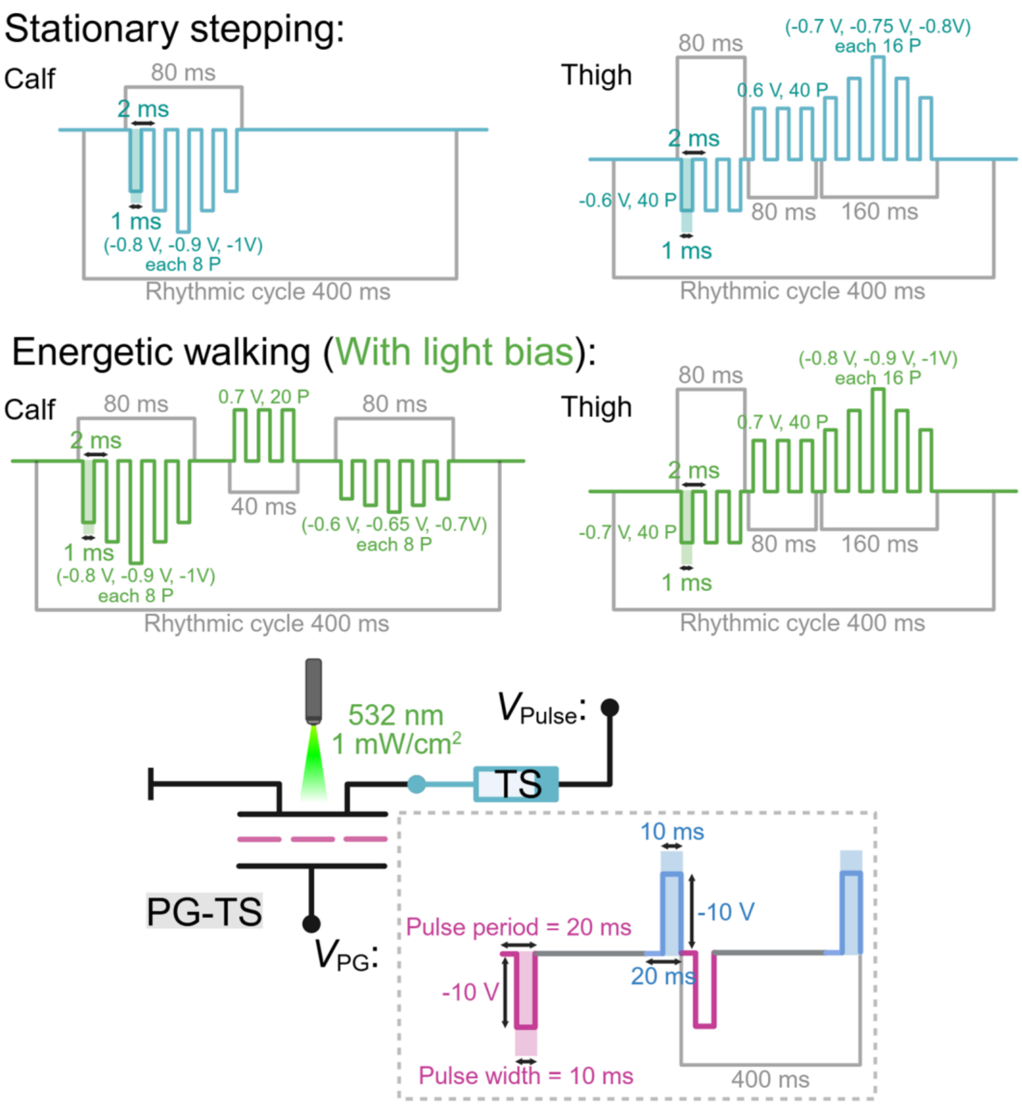

Fig. R2.4 Control voltages waveform for the three-phase locomotion. V_{PG} is a periodic depolarizing/hyperpolarizing voltage with 50% duty cycle and an amplitude of 10 V. Compared to steady slow walking, the stationary-stepping phase lacks the reverse-oscillation input stage and the small-amplitude staircase input stage for calf. Leveraging 2D optical response of the plateau neurons, a visible-light (532 nm) bias of 1 mW/cm^2 applied under the pulse conditions for slow walking. The rhythmic spikes output exhibit photo-adaptive enhancement, thereby meeting the large-amplitude swing required for the robot's energetic walking.

Text Revision:

Based on the reviewer's helpful suggestion to the quadruped walks, we revise the manuscript accordingly on page 8, page 21:

We add related discussion to the manuscript on page 8, lines 291-303:

Beyond steady-state operation, we implement adaptive walking to demonstrate the tunable capability of the plateau neuron hardware. By modulating input voltage to the plateau neuron circuits and enhancing the rhythmic spiking under optical bias (Supplementary Fig. 20), we command the robot to execute a three-phase adaptive sequence: stationary stepping, cautious walking (0.15 m/s), and energetic walking (0.4 m/s) (Fig. 5e) (Supplementary Video 4). The resulting adaptive rhythmic spike bursts, which maintain the 200-ms phase difference between left and right limbs, are shown in Fig. 5f. The smoothly transitioning joint-angle profiles (Fig. 5g) and the evolving spatiotemporal heatmap of spike bursts (Fig. 5h) illustrate the system's capacity for continuous behavioral modulation based on external inputs. This demonstrates that the *in-situ* spike malleability of plateau neurons harnessed for dynamic gait adaptation. Taken together, these experiments provide evidence that our neuromorphic CPG hardware can generate control signals sufficient for effective on-ground locomotion and exhibits the inherent programmability required for adaptive behavior.

We add the supplementary experimental results figure and corresponding caption in Fig5 on page 20, lines 620–631:

We present the expanded Abstract in the main text on page 1, lines 30–32:

Critically, the system achieves stable on-ground locomotion and demonstrates adaptive gait transitions in real-world environments.

We revise the wording in the Introduction on page 3, lines 87–90:

We validate stable trotting through simulations (Isaac Gym) and physical experiments, including on-ground walking and adaptive gait modulation via external inputs, with programmable spiking dynamics enabling rhythmic locomotion.

We add text to the Discussion on page 9, lines 324–327:

The system successfully executes stable on-ground locomotion and exhibits adaptive walking through direct modulation of plateau neuron's operating point, confirming its potential for real-world deployment in dynamic environments.

The supporting information is revised accordingly on page 23:

We add Supplementary Figure 20 titled: "Control voltages waveform for the three-phase locomotion." on page 23, lines 221-228:

(**Supplementary Figure 20** here refers to **Fig. R2.4** mentioned above and would not be listed again.)

Comment #4: "The paper should provide a more comprehensive analysis of energy efficiency at the system level such as details regarding motor driving current, torque output, total energy consumption, and direct comparisons during actual locomotion. While the device demonstrates impressive energy efficiency at the chip level, the study

does not yet establish that this translates into overall energy savings for the full quadrupedal locomotion system. Providing quantitative data or benchmarking against existing robotic control architectures would also strengthen the claim of system-level energy improvement."

Our Response:

We thank the reviewer for the suggestion to perform a comprehensive system-level energy analysis. This significantly strengthens our manuscript. In response, we conduct new measurements and analyses, which collectively demonstrate the energy advantage of our neuromorphic control approach at both the subsystem and full-system levels.

Full-System Power Measurement and Comparison: We measure the total motor-drive power of the Unitree Go2 robot during stable on-ground walking at 0.4 m/s, as requested. The real-time power profile and the calculated average power of 219.7 W are presented in Fig. R2.6 a. To provide a fair benchmark, we implement an identical walking gait using a conventional software-based PD controller running on a microcontroller unit (MCU) and measure its total motor-drive power under the same conditions. The comparison (Fig. R2.6 b) shows that our PG-TS-based control system reduces the total motor-drive power by 13.64% (from 254.4 W to 219.7 W).

Ultra-Low Overhead of the Neuromorphic Control Core: The system-level saving originates from the extreme efficiency of the control signal generator itself. We measure the active power of the PG-TS circuits during operation. At full load, the average power per PG-TS core driving a joint is only ~1.25 μW. The estimated total power for the eight cores controlling all eight active joints is thus merely ~10 μW (see Fig. R2.6 b legend).

Torque Output and Scaling Analysis: We also supplement the requested torque data for major joints during walking (Fig. R2.5). More importantly, to address the reviewer's concern about peripheral overhead in a complete system, we provide a scaling analysis (Fig. R2.6 c). It illustrates that in a scale-up system (as envisioned in Supplementary Fig. 19), the fixed power of shared peripheral circuits is amortized over

many PG-TS cores. The total control system power then scales approximately linearly with the core count, governed by its ultra-low per-core base (~1.25 μW/core). This establishes a favorable scaling trajectory compared to digital architecture.

In summary, these new results demonstrate that our neuromorphic approach (1) reduces full-system energy consumption for real-world quadrupedal locomotion, (2) achieves this through a fundamentally different paradigm—a μW scale "Rhythm Engine" that replaces digital computation for core pattern generation, and (3) is built on a scalable architecture primed for greater efficiencies at larger scales. Together, they substantiate the pathway from device-level innovation to practical system-level advantage.

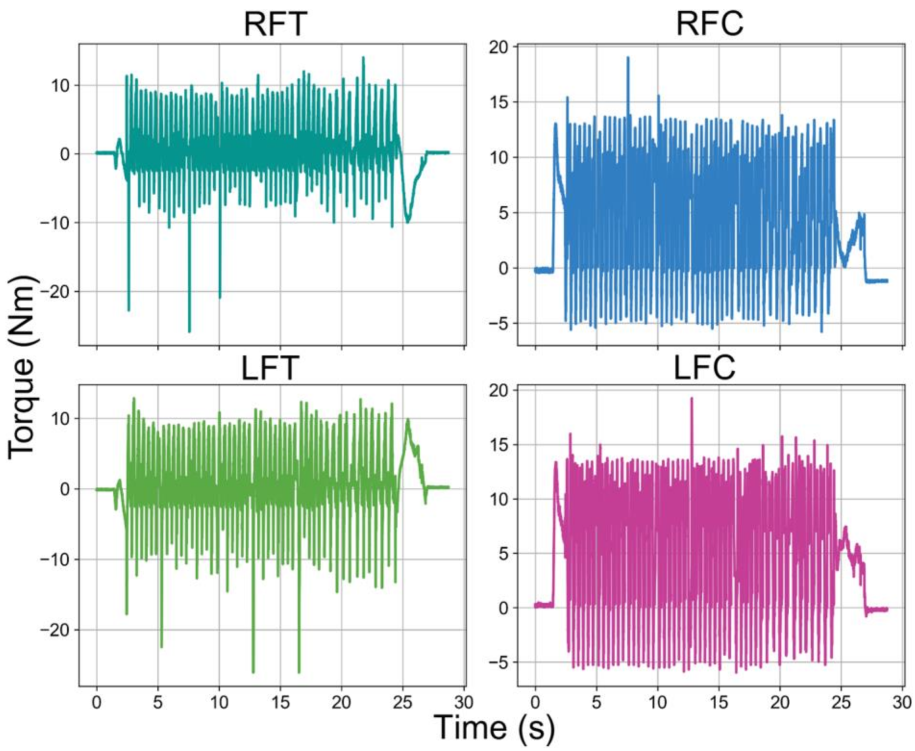

Fig. R2.5 Measured joint torque during energetic walking of the Unitree Go2 robot. The plot records the torque outputs from the four key actuation joint groups—RFT, RFC, LFT, and LFC—during a stable walking phase at 0.4 m/s. The data indicate that the calf joints generate larger torque amplitudes to propel the faster forward stepping characteristic of the energetic gait.

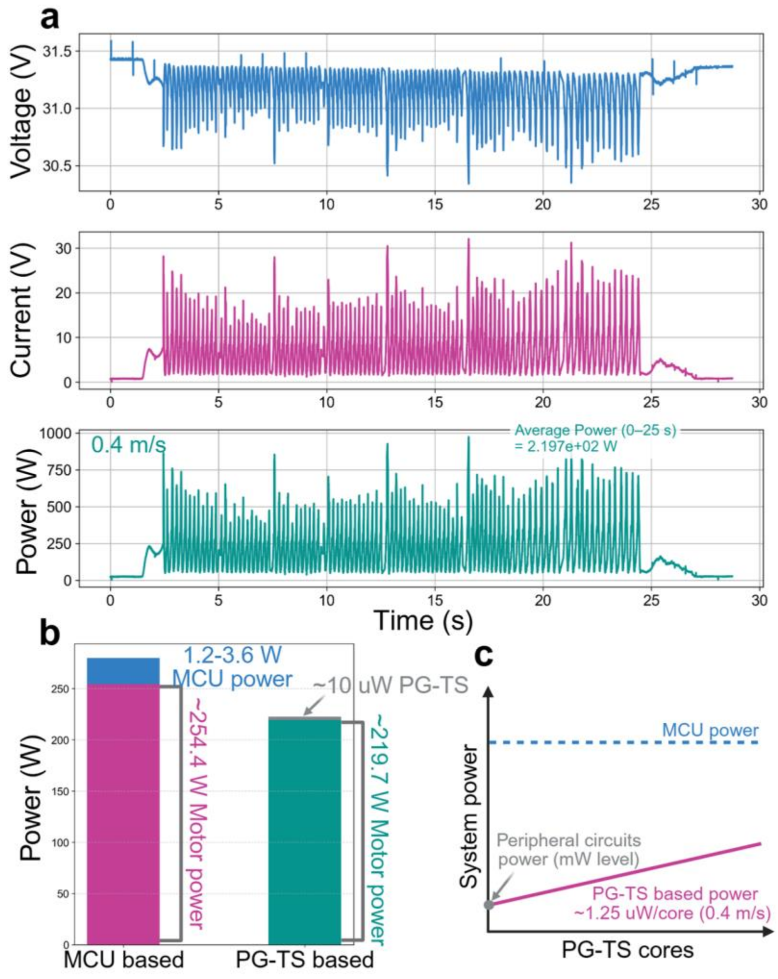

Fig. R2.6 System-level power analysis of quadrupedal walking. a, The real-time total power consumption of all joint motors on the Unitree Go2 robot during energetic walking, calculated from the recorded driving voltages and currents. The average power over the 0-25 s period is 219.7 W. b, A direct comparison of the average motor-drive power under two control paradigms for the same robot and gait condition. Conventional MCU-based software control consumes approximately 254.4 W (in the absence of algorithmic power optimization). In contrast, the PG-TS-based neuromorphic hardware control reduces the power to 219.7 W, representing a 13.64% decrease. The PG-TS circuits operate at an ultra-low power: under full load (V_{Pulse} = 1.0 V), the average spiking current is 1.24688 μA, leading to a per-core power of ~1.25 μW. The total estimated power for the eight PG-TS cores driving all joints is thus ~10 μW (not yet including fixed peripheral overhead). c, Scaling analysis of the system power. While the total motor power is dominant, the neuromorphic control circuitry power scales

favorably. As conceptualized in Supplementary Fig. 19, in a scaled system with many PG-TS cores sharing fixed peripheral circuits, the total control system power increases approximately linearly with the number of cores. This contrasts with digital architecture where frequent data movement and computation incur high dynamic power, highlighting the efficiency potential of the in-memory, analog rhythmic generation paradigm[2].

Text Revision:

Based on the reviewer's helpful suggestion to the energy efficiency at system level, we revise the manuscript accordingly on page 8:

We add related discussion to the manuscript on page 8, lines 286-290:

We further evaluate the system-level energy efficiency of this neuromorphic control approach. The joint torque profiles during walking are provided in Supplementary Figure. 18. A comprehensive power analysis (Supplementary Fig. 19) shows that during steady walking at 0.4 m/s, the PG-TS-based system reduces the total motor-drive power by 13.64 % compared to a conventional microcontroller unit (MCU)-based PD controller.

The supporting information is revised accordingly on page 21, page 22:

We add Supplementary Figure 18 titled: "Measured joint torque during energetic walking of the Unitree Go2 robot." on page 21, lines 199-203:

(**Supplementary Figure 18** here refers to **Fig. R2.5** mentioned above and would not be listed again.)

We add Supplementary Figure 19 titled: "System-level power analysis of quadrupedal walking." on page 22, lines 205-220:

(**Supplementary Figure 19** here refers to **Fig. R2.6** mentioned above and would not

be listed again.)

Comment #5: "What specific features of the proposed circuit necessitate a custom fabrication process? From a practical standpoint, could this design be implemented using standard mixed-signal processes, such as TSMC 130 nm. Additionally, the authors should elaborate on how they envision deploying this technology in practical robotic systems: as standalone neuromorphic chips or integrated modules within existing control IC."

Our Response:

We thank the reviewer for raising these critical questions regarding fabrication and practical deployment, which allow us to clarify the rationale for our custom platform and its integration pathway.

The unique computational functionalities of the PG-TS device—specifically, its bistable plateau potential and in-situ spike malleability—are intrinsically enabled by its material stack. The non-volatile, analog modulation of the MoS_2 channel via the graphene floating gate is fundamental to generating the latching plateau behavior. This monolithic structure concurrently provides embedded photosensitivity, as utilized in our adaptive demonstrations. Coupled with the Ag-filament-based transient switch for leaky LIF dynamics, this compact physics-based design achieves core neuromorphic functions within a single, ultra-efficient unit. Implementing equivalent analog memory, transient switching, and sensory response in a standard CMOS process would necessitate a complex multi-transistor circuit, thereby forfeiting the area and power advantages central to our approach[6]. Such an implementation would inevitably erode the core advantages of area efficiency and ultra-low power consumption that define our physics-based, monolithic device. Therefore, the present custom 2D hetero-integration platform is essential for exploring this bio-inspired computational primitive in its most optimized form.

We acknowledge the reviewer's concern regarding practical deployment and

envision a pragmatic pathway forward through heterogeneous integration. This aligns with established trends in advanced semiconductor systems, aiming not to replace silicon electronics but to augment them with specialized, ultra-efficient "engines." In this vision, the analog PG-TS core array can be fabricated as a dedicated, ultra-low-power "Rhythm Generation and Fast Adaptation Unit." This unit can subsequently be integrated via mature packaging-level technologies (e.g., using 2.5D interposers or 3D system integration) with a standard silicon CMOS chip responsible for higher-level decision-making, communication, and complex algorithms[4,5]. This "best-of-both-worlds" architecture allows each technology to excel in its respective role: our neuromorphic core delivers unparalleled efficiency for low-level rhythmic control and fast sensorimotor loops, while mature digital logic handles the complex computations for which it is best suited. This integration strategy, grounded in current industry capabilities, represents a clear and feasible path to application.

Text Revision:

Based on the reviewer's helpful suggestion, we revise the manuscript accordingly on page 9:

We add related discussion to the manuscript on page 9, lines 330-332:

For practical deployment, a viable path lies in heterogeneous integration, where such neuromorphic cores can be combined with digital silicon CMOS.

Comment #6: "Could you provide benchmarking of the reported activation latency of 5.62 μs against existing neuromorphic or conventional control circuits?"

Our Response:

We thank the reviewer for this constructive suggestion to benchmark our activation latency. In response, we conduct a comprehensive comparative analysis and add a new Table R2.1, titled "Comparison of activation latency and related performance metrics

for robotic control”. This table situates our work within the broader landscape of recent neuromorphic and bio-inspired control systems, encompassing a spectrum from software-centric to full-hardware implementations.

The comparison reveals several key insights that contextualize the performance advantage of our architecture:

Superior Activation Latency for Hardware CPGs: Among hardware implementations targeting rhythmic locomotion control (CPG function), our reported activation latency of **5.62 μs** stands out. It is orders of magnitude faster than other coupled-oscillator CPG implementations (e.g., ~ms-scale for VO_2-based systems [Bonagiri et al., Dutta et al.]) and software-based SNN controllers (~10 ms [Hashemkhani et al.]). This microsecond-level response time is a direct benefit of our compact, analog 2(PG-TS) circuit design.

Balanced Performance Profile: While a specialized reflex circuit [NbO_2 SCNC] demonstrates even lower latency (100 μs) for reactive obstacle avoidance—a task with different temporal constraints—our work achieves a unique combination of low latency (5.62 μs), high energy efficiency (141.37 pJ/spike), and rich neuronal dynamics (rhythmic bursts with in-situ malleability). This balance is tailored for the core task of stable, programmable rhythm generation.

Practical Significance for Locomotion: The activation latency requirement for stable legged locomotion is governed by mechanical time constants. A control latency in the range of 10-100 ms (10-100 Hz) is widely considered sufficient and standard for dynamic gait generation in mid-sized quadruped[7,8]. Our achieved latency of 5.62 μs translates to a potential control bandwidth far exceeding this requirement, providing a substantial performance margin. This margin is not merely incremental; it is a concrete enabler for future advancements requiring ultra-high bandwidth control, such as dynamic balance recovery on uneven terrain or high-speed galloping.

Holistic Validation: Crucially, this low-latency performance is not a theoretical projection but is integral to the system that successfully demonstrated physical quadruped trotting (as responding to Comment 2), bridging the gap from device physics to real-world robotic function.

In summary, the benchmarking confirms that our PG-TS-based CPG architecture occupies a unique and advantageous position: it delivers the speed necessary for high-performance real-time control while maintaining the ultra-low energy consumption and biological fidelity essential for next-generation autonomous robotics.

Table R2.1 Comparison of activation latency and related performance metrics for robotic control

	**Hashemkhani et al.[9]**	**Bonagiri et al.[10]**	**Yang et al.[11]**	**Chen et al.[12]**	**Dutta et al.[13]**	**This work**
Architecture	Hierarchical SNN with DSM	VO_2 IMT-O	NbO_2 SCNC	Mixed tactile SNN	VO_2 IMT-NO	2(PG-TS)
Activation Latency	~10 ms	~ms scale	~100 µs	~1.2 ms	~83 ms	~5.62 µs
Neuronal Dynamics	Rhythmic spike bursts	Relaxation oscillation	Mixed spiking/bursting	LIF spiking	Relaxation oscillation	Rhythmic spike bursts
Modulation	Phase	Phase	Spiking-bursting transition	Feature extraction	Phase	Amplitude, phase, frequency
Energy per Spike	68 mJ/event	-	1.06 pJ (theor.)	72 nJ	2.67 µJ	141.37 pJ
Process Technology Implementation	Software (Raspberry Pi/Arduino)	Hybrid (VO_2 + CMOS model)	Hybrid (NbO_2 + R/C on PCB)	Hybrid (TENG + CMOS)	Hybrid (VO_2 + CMOS)	Wafer-scale 2D heterostructure
Application Demonstrated	Quadruped obstacle avoidance	Six quadruped gaits (sim.)	Robotic obstacle avoidance	Object classification	Quadruped gaits (sim.)	Quadruped trotting (sim. & robot)

SNN Spiking neural network, DSM dynamic state machine, IMT insulator-to-metal phase transition, NO nano oscillators, SCNC sensorimotor control neural circuit, CMOS complementary metal oxide semiconductor, R/C Resistance/Capacitance, TENG triboelectric nanogenerator.

<u>Text Revision:</u>

Based on the reviewer's helpful suggestion to benchmark our activation latency, we revise the manuscript accordingly on page 5:

We add related discussion to the manuscript on page 5, lines 157-162:

Among hardware implementations for rhythmic locomotion, our latency of 5.62 μs is notably lower than other oscillator-based CPGs (e.g., VO_2-based systems operating at ms-scale) and software-based spiking network controllers (~10 ms) (Supplementary Table 1). This microsecond-level response provides a substantial performance margin well within the 10-100 ms control latency requirement for stable dynamic gait locomotion, enabling future exploration of high-bandwidth adaptive control.

The supporting information is revised accordingly on page 25, page 26:

We add Supplementary Table 1 titled: "Comparison of activation latency and related performance metrics for robotic control" on page 25:

(**Supplementary Table 1** here refers to **Table R2.1** mentioned above and would not be listed again.)

We add citations to Supplementary Table 1 on page 26, line 253-264:

4 Kudithipudi, D. *et al.* Neuromorphic computing at scale. *Nature* **637**, 801-812, doi:10.1038/s41586-024-08253-8 (2025).

5 Hashemkhani, S., Vivekanand, V. S., Chopra, S. & Kubendran, R. Toward autonomous event-based sensorimotor control with supervised gait learning and obstacle avoidance for robot navigation. *Front Neurosci* **19**, 1492436 (2025).

6 Bonagiri, A., Biswas, D. & Chakravarthy, S. Coupled Memristor Oscillators for Neuromorphic Locomotion Control: Modeling and Analysis. *IEEE Trans Neural Netw Learn Syst* **35**, 8638-8652 (2024).

7 Yang, Y. *et al.* Firing feature-driven neural circuits with scalable memristive neurons for robotic obstacle avoidance. *Nat Commun* **15**, 4318 (2024).

8 Chen, L. *et al.* Spike timing-based coding in neuromimetic tactile system enables dynamic object classification. *Science* **384**, 660-665 (2024).

Comment #7: "(Minor) In Table 1, the unit for biological neurons in the first column should be corrected from "pj" to "pJ.""

Our Response:

We sincerely apologize for this oversight. The unit in Table 1 correct to "pJ" as suggested.

Text Revision:

Based on the reviewer's helpful suggestion, we revise Table 1 on Page 21 accordingly. In the updated manuscript, the unit in the *Energy/spike* for biological neurons (first column) correct from "pj" to "pJ".

Reference:

1 Kudithipudi, D. *et al.* Neuromorphic computing at scale. *Nature* **637**, 801-812 (2025).

2 Wan, W. *et al.* A compute-in-memory chip based on resistive random-access memory. *Nature* **608**, 504-512 (2022).

3 Lee, J. *et al.* BEOL-Compatible Non-Volatile Capacitive Synapse with ALD W-Doped In2O3 Semiconductor Layer. in *2024 IEEE International Electron Devices Meeting (IEDM)* 1-4 (2024).

4 Xie, Y. *et al.* First Demonstration of 2.5D Out-of-Plane-Based Hybrid Stacked Super-Bionic Compound Eye CMOS Chip with Broadband (300–1600 nm) and Wide-Angle (170°) Photodetection. in *2024 IEEE International Electron Devices Meeting (IEDM)*1-4 (2024).

5 Liu, C. *et al.* A full-featured 2D flash chip enabled by system integration. *Nature* **646** (2025).

6 Zhu, Z. *et al.* Recent advances in spike-based neural coding for tactile perception.

Microsystems & Nanoengineering **11** (2025).

7 Hwangbo, J. *et al.* Learning agile and dynamic motor skills for legged robots. *Sci Robot* **4**, 5872 (2019).

8 Humphreys, J. & Zhou, C. Learning to adapt through bio-inspired gait strategies for versatile quadruped locomotion. *Nature Machine Intelligence* **7**, 1141-1153 (2025).

9 Hashemkhani, S., Vivekanand, V. S., Chopra, S. & Kubendran, R. Toward autonomous event-based sensorimotor control with supervised gait learning and obstacle avoidance for robot navigation. *Front Neurosci* **19**, 1492436 (2025).

10 Bonagiri, A., Biswas, D. & Chakravarthy, S. Coupled Memristor Oscillators for Neuromorphic Locomotion Control: Modeling and Analysis. *IEEE Trans Neural Netw Learn Syst* **35**, 8638-8652 (2024).

11 Yang, Y. *et al.* Firing feature-driven neural circuits with scalable memristive neurons for robotic obstacle avoidance. *Nat Commun* **15**, 4318 (2024).

12 Chen, L. *et al.* Spike timing-based coding in neuromimetic tactile system enables dynamic object classification. *Science* **384**, 660-665 (2024).

13 Dutta, S. *et al.* Programmable coupled oscillators for synchronized locomotion. *Nat Commun* **10**, 3299 (2019).
